# Epigenetic resetting in the human germ line entails histone modification remodeling

**DOI:** 10.1126/sciadv.ade1257

**Published:** 2023-01-18

**Authors:** Wolfram H. Gruhn, Walfred W.C. Tang, Sabine Dietmann, João P. Alves-Lopes, Christopher A. Penfold, Frederick C. K. Wong, Navin B. Ramakrishna, M. Azim Surani

**Affiliations:** ^1^ Wellcome Trust/Cancer Research UK Gurdon Institute, Henry Wellcome Building of Cancer and Developmental Biology, Cambridge CB2 1QN, UK.; ^2^ Physiology, Development and Neuroscience Department, University of Cambridge, Cambridge CB2 3EL, UK.; ^3^ Wellcome–MRC Cambridge Stem Cell Institute, Jeffrey Cheah Biomedical Centre, Puddicombe Way, Cambridge Biomedical Campus, Cambridge CB2 0AW, UK.; ^4^ Institute for Informatics, Washington University School of Medicine, St. Louis, MO, USA.; ^5^ NORDFERTIL Research Lab Stockholm, Childhood Cancer Research Unit, J9:30, Department of Women’s and Children’s Health, Karolinska Institutet and Karolinska University Hospital, Visionsgatan 4, 17164, Solna, Stockholm, Sweden.; ^6^ Centre for Trophoblast Research, University of Cambridge, Cambridge, UK.; ^7^ Genome Institute of Singapore, A*STAR, Biopolis, Singapore 138672, Singapore.

## Abstract

Epigenetic resetting in the mammalian germ line entails acute DNA demethylation, which lays the foundation for gametogenesis, totipotency, and embryonic development. We characterize the epigenome of hypomethylated human primordial germ cells (hPGCs) to reveal mechanisms preventing the widespread derepression of genes and transposable elements (TEs). Along with the loss of DNA methylation, we show that hPGCs exhibit a profound reduction of repressive histone modifications resulting in diminished heterochromatic signatures at most genes and TEs and the acquisition of a neutral or paused epigenetic state without transcriptional activation. Efficient maintenance of a heterochromatic state is limited to a subset of genomic loci, such as evolutionarily young TEs and some developmental genes, which require H3K9me3 and H3K27me3, respectively, for efficient transcriptional repression. Accordingly, transcriptional repression in hPGCs presents an exemplary balanced system relying on local maintenance of heterochromatic features and a lack of inductive cues.

## Teaser

Rejuvenating the human germline for the next life cycle involves profound epigenetic remodelling.

## INTRODUCTION

**Fig. 1. F1:**
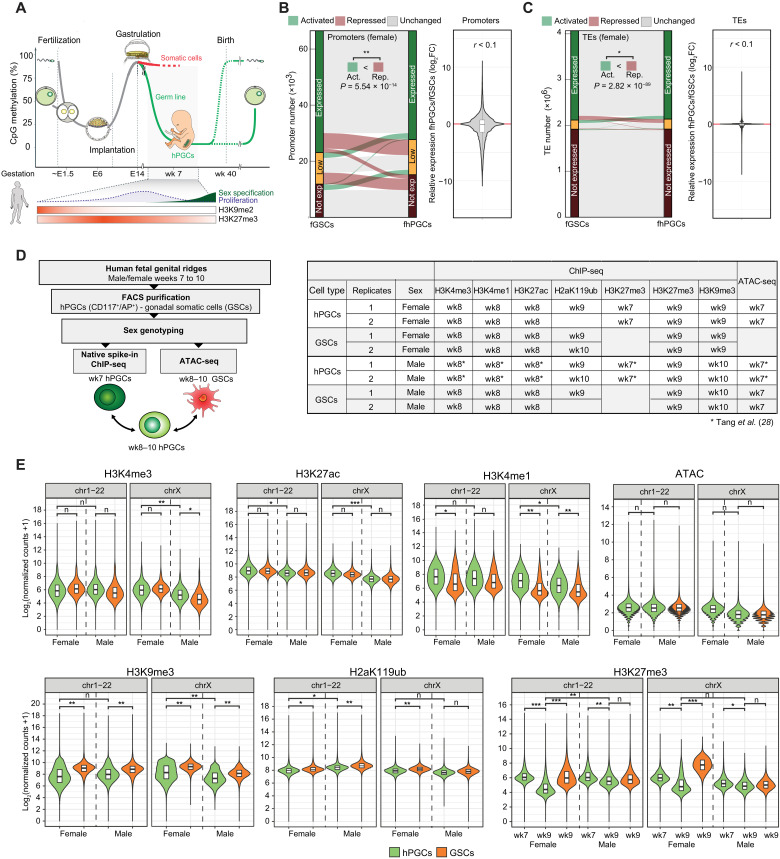
Epigenetic resetting in hPGCs coincides with a reduction of repressive histone modifications. (**A**) Graphical depiction of 5mC changes during major epigenetic remodeling events in the human life cycle. Previously described changes of H3K27me3 and H3K9me2 during hPGC maturation are as shown. (**B** and **C**) Comparison of protein-coding and long noncoding RNA gene (B) and TE (C) expression between fhPGCs and fGSCs. Genes/TEs were grouped according to their expression level into expressed (dark green), lowly expressed (orange), and not expressed genes (dark red; see Materials and Methods). No broad expression change (gray), reduced expression (red), or elevated expression (green) in fhPGCs (left). Wilcoxon effect size and chi-square test for number of induced and repressed elements are shown. Violin plot depicting expression changes of 66,495 human transcripts (B) and 3,040,811 annotated TEs (C) in fhPGCs relative to fGSCs (right). *r* = Wilcoxon effect size. log_2_FC, log_2_ fold change. (**D**) Graphical depiction of the spike-in normalized histone modification ChIP-seq and ATAC-seq conducted on weeks 7 to 10 hPGCs and GSCs (right). Used tissues and analyzed histone modifications are indicated (right table). ATAC-seq and H3K27me3 (wk7), H3K4me1, H3K4me3, and H3K27ac on male hPGCs have been published in Tang *et al.* (*28*). (**E**) Spike-in normalized read counts falling into 5-kb bins covering all autosomes and X chromosomes for the indicated ChIP-seq experiments on male and female PGCs and GSCs. ATAC-seq reads were analyzed in 1-kb bins. Wilcoxon effect size is shown. Effect size levels: no practical difference (n): *r* < 0.2, *: 0.2 ≤ *r* < 0.3, **: 0.3 ≤ *r* < 0.5, ***: *r* ≥ 0.5.

Germ line development is an integral part of the life cycle, culminating in the formation of gametes, which transfer genetic and epigenetic information to the next generation. Human primordial germ cells (hPGCs), the embryonic precursors of sperm and egg, originate during the onset of gastrulation between week (wk) 2 and 3 of development and thereafter commence migration via the hindgut mesentery to the embryonic gonads at ~wk4, where hPGCs undergo sex-specific differentiation (*1*). The migration of hPGCs into the developing gonad is accompanied by genome-wide epigenetic changes, including a profound loss of DNA methylation (*2*), which is critical for erasing genomic imprints, promoting PGC-specific gene expression and X chromosome reactivation. Consequently, epigenetic resetting in the early germ line is critical for gametogenesis and embryonic development in the next generation (*3*–*5*). Evidence from several mammalian species suggests that epigenetic resetting in the germ line extends to changes in repressive histone modifications such as H3K27me3 (Fig. 1A) (*6*–*10*).

The polycomb repressive complex 1 (PRC1) and PRC2 that deposit the H2aK119ub and H3K27me3 histone modifications, respectively, mediate transcriptional repression (*11*) during X chromosome inactivation (*12*) and at autosomal promoters with high cytosine-guanine (CG) density, which often control developmentally relevant genes (*13*). The histone modification H3K9me3 mediates transcriptional repression of olfactory receptors (*14*) and testis-associated genes (*15*), while in other contexts, promoter-proximal H3K9me3 is required for normal gene transcription (*16*). In addition, H3K9me3 and its histone methyltransferase SETDB1 are widely implicated in repressing transposable elements (TEs) (*15*) and cryptic promoters to permit normal gene expression (*17*).

Approximately 50% of the human genome originates from TE insertions (*18*), with the vast majority having lost the potential for transposition (*19*). Some TEs have been co-opted for host gene regulatory functions (*20*). Exceptionally, some TEs belonging to evolutionarily young families, such as the primate-specific SINE-VNTR-Alu (SVA) elements, remain competent for autonomous transcription and require tight repression involving H3K9me3 and DNA methylation to maintain genome stability (*21*). However, virtually all promoters and TEs that are methylated in somatic cells exhibit reduction or loss of DNA methylation (5mC) during epigenetic resetting in hPGCs (*2*). Nonetheless, only a small fraction of TEs gains transcriptional activity in hPGCs; however, how transcriptional repression is maintained in the hypomethylated germ line is not fully understood (*15*, *22*).

In this study, we investigate the epigenome of hypomethylated male and female hPGCs (mhPGCs and fhPGCs) and gonadal somatic cells (mGSCs and fGSCs) by integrating repressive (H3K27me3, H2aK119ub, and H3K9me3), neutral (H3K4me1), and active (H3K4me3 and H3K27ac) histone modification, chromatin accessibility, transcription and DNA methylation profiles to gain insights into transcriptional regulation in hPGCs. Compared to GSCs, reset hPGCs exhibited lower levels of 5mC and repressive histone modifications, consistent with a broadly weakened heterochromatic state of promoters and TEs. Notably, specific TE and gene groups such as SVAs or olfactory receptor genes (ORGs) were particularly enriched for maintaining a repressive chromatin state in hPGCs. Our functional studies suggest that the H3K9me3-SETDB1 axis is critical for TE repression and the timing of gene activation in hPGCs. Furthermore, the gain of H3K4me1 at demethylated promoters and the retention of repressive H3K27me3 at promoter-proximal TEs might contribute to gene repression in hPGCs.

## RESULTS

### Global epigenetic differences between hPGCs and surrounding somatic cells

The best understood the aspect of epigenetic resetting in the human germ line is the loss of DNA methylation, which is initiated before the fifth week of embryogenesis and largely completed in wk7 gonadal hPGCs (Fig. 1A) (*2*). In addition, immunofluorescence analysis suggests that the levels of repressive histone modifications such as H3K9me2 and H3K27me3 change during the resetting process (*2*). Consistently, we found decreasing H3K27me3 levels between wk7 and wk9 in fluorescence-activated cell sorting (FACS)–purified hypomethylated fhPGCs by Western blotting (fig. S1, A and B). Despite these profound epigenetic changes, reanalysis of published RNA sequencing (RNA-seq) data (*2*) of hypomethylated germ cells did not detect genome-wide derepression of either genes (Fig. 1B and fig. S1C) or TEs (Fig. 1C and fig. S1D) relative to the adjacent GSCs.

A similar loss of DNA methylation in migrating and early gonadal PGCs occurs in other mammalian species, including mice, pigs, and rabbits (*23*–*25*). However, cellular levels of H3K27me3 and H2aK119ub in wk8/9 hPGCs were substantially lower than that in murine PGCs at a similar developmental stage, suggesting interspecies differences in the germ line resetting process (fig. S1E).

We, therefore, set out to investigate how histone modifications might contribute locus specifically to transcription repression in the hypomethylated human germ line. An in-depth epigenetic analysis of hPGCs before epigenetic resetting is not feasible for technical and ethical reasons. Consequently, we focused on hypomethylated gonadal hPGCs and the adjacent GSCs, which do not undergo genome-wide DNA demethylation while experiencing a similar morphogen environment to hPGCs and therefore represent a reference point to identify putative germ line-specific gene regulatory mechanisms. To this end, we adapted the ultra-low-input micrococcal nuclease (MNase)-based native chromatin immunoprecipitation (ULI-NChIP) protocol to analyze key repressive and active histone modifications in purified hPGCs and GSCs and probed chromatin accessibility by transposase-accessible chromatin sequencing (ATAC-seq; Fig. 1D and fig. S1A) (*26*). Here, we introduced *Drosophila* S2 cell spike-ins to accurately normalize histone modification levels in the different cell types (*27*). We focused on wk7 to wk10 male and female genital ridges as germ line DNA demethylation is largely completed at these stages, and sufficient numbers of hPGCs can be isolated to reliably prepare one ULI-NChIP library per embryo (fig. S1, F and G) (*2*). We have previously described a portion of these data [ATAC-seq in mhPGCs and H3K27m3 (wk7), H3K4me3, H3K4me1, and H3K27ac ChIP-seq (ChIP sequencing) in mhPGCs] in a different context (*28*).

First, we determined the global levels of specific histone modifications at autosomes and sex chromosomes separately to avoid biases due to the reactivation of the inactive X chromosome (Xi) in fhPGCs (Fig. 1E and fig. S1H). H3K4me1 levels at autosomes and sex chromosomes showed substantial enrichment in mhPGCs and fhPGCs relative to GSCs, consistent with elevated H3K4me1 levels detected in gonadal hPGCs by immunofluorescence staining (fig. S1I). In contrast, there was a reduction in repressive histone modification levels, H3K27me3, H2aK119ub, and H3K9me3 in hPGCs compared to somatic cells at wk8 to wk10 of development. Consistent with the ChIP-seq results, lower H2aK119ub levels in wk9 hPGCs than in GSCs were detected by Western blot (fig. S1E). Notably, there was a more pronounced reduction in H3K27me3 levels in fhPGCs between wk7 and wk9 than in mhPGCs (Fig. 1E).

### Sex-specific epigenetic promoter regulation in hPGCs

Between wk5 and wk9, mhPGCs and fhPGCs are mitotically active and transcriptionally highly similar, with sexual differentiation occurring later in development (*29*, *30*). Hence, the epigenetic differences between sexes observed in wk9 hPGCs (Fig. 1) could prime sexual differentiation, which prompted us to compare the epigenetic states of 64,189 autosomal promoters in both sexes.

**Fig. 2.**
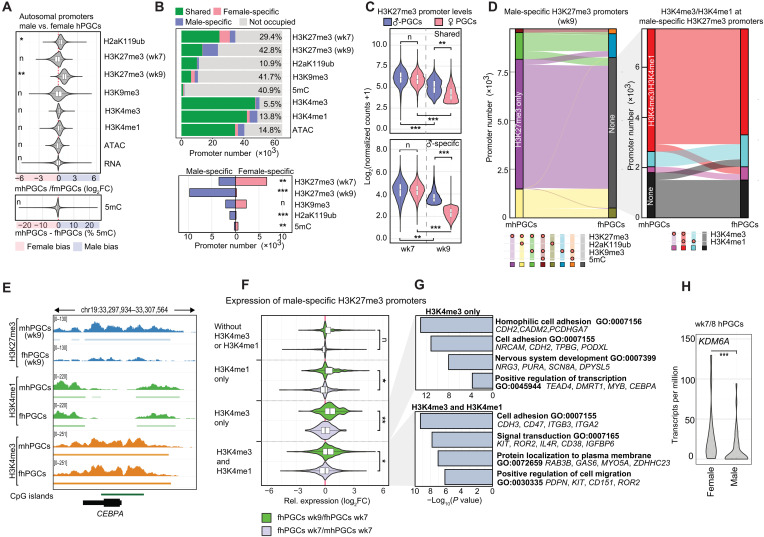
Epigenetic promoter regulation in male and female hPGCs. (**A**) Differential levels of the indicated epigenetic marks, chromatin accessibility (ATAC), and transcriptional activity (RNA) of autosomal promoters in male and female hPGCs are depicted. Wilcoxon effect size is depicted. (**B**) Top: Number of promoters occupied with the indicated epigenetic modifications or ATAC-seq signal. Male-specific occupancy (blue), female-specific occupancy (red), occupancy in both sexes (green), and no occupancy (gray). Sex-specifically occupied promoter portion is indicated. Bottom: Number of male- (blue) and female-specific (red) promoters occupied by the indicated repressive chromatin marks. Chi-square goodness-of-fit effect size is depicted. (**C**) H3K27me3 levels of promoters commonly occupied by H3K27me3 in wk9 male and female hPGCs (top) and male-specifically H3K27me3 occupied promoters in wk9 hPGCs (bottom). Wilcoxon effect size is depicted. (**D**) Epigenetic states of promoters specifically occupied by H3K27me3 in mhPGCs (wk9) and the corresponding promoter states in wk9 fhPGCs (left). Distribution of H3K4me3 and H3K4me1 is shown for promoters occupied by H3K27me3 in mhPGCs but harbors no repressive mark in fhPGCs (right). (**E**) Genome browser view of the *CEBPA* locus showing the indicated epigenetic modifications in fhPGCs and mhPGCs. (**F**) Differential expression (DE) of genes associated with male-specific H3K27me3 promoters co-occupied by the indicated epigenetic marks between wk7 and wk9 fhPGCs (green) and wk7 mhPGCs and fhPGCs (light blue). Wilcoxon effect size is depicted. (**G**) Gene ontology (GO) enrichment analysis on promoter groups depicted in Fig. 2F. (**H**) *KDM6A* expression in mhPGCs and fhPGCs. Reanalysis of single-cell RNA-seq (scRNA-seq) data. Sleuth’s likelihood ratio test is shown. ***P < 0.005. Effect size levels as in Fig. 1.

In line with our genome-wide analysis, male-biased autosomal promoter occupation was detected for H2aK119ub and H3K27me3 in wk9 hPGCs. This was not the case for other epigenetic marks or H3K27me3 in wk7 hPGCs (Fig. 2A). The portion of promoters showing sex-specific occupancy varies among epigenetic marks between 42.75% for H3K27me3 (wk9) and 5.51% for H3K4me3 (Fig. 2B). Sex-specific promoter occupancy was prominent for H3K27me3, H2aK119ub, and H3K9me3, with H3K27me3 in wk9 hPGCs showing the strongest overrepresentation of male-specifically occupied promoters. Both male- and female-specific H3K9me3 occupied promoters showed strong enrichment for ORGs, which were flanked or covered by H3K9me3 domains that showed slight sex-specific positional shifts (fig. S2, A and B). Nevertheless, we detected no sex-specific expression of these ORGs when reanalyzing published bulk (*2*) or single-cell RNA-seq (scRNA-seq) data (*30*) of wk7/8 hPGCs (fig. S2C). Some genes associated with the Piwi-interacting RNA (piRNA) pathway, e.g., *PIWIL2*, were preferentially occupied by H3K9me3 in fhPGCs but showed increased expression in mhPGCs, supporting an H3K9me3-mediated transcriptional regulation of these genes (fig. S2, D and E).

Between wk7 and wk9, H3K27me3 promoter occupancy decreased in both fhPGCs and mhPGCs, which was more pronounced in fhPGCs resulting in 41.7% of all H3K27me3 promoters being male-specifically occupied in wk9 hPGCs (Fig. 2, B and C). In mhPGCs, 76.0% of male-specific H3K27me3 promoters were co-occupied by H3K4me3 alone or in combination with H3K4me1, while the corresponding promoters in fhPGCs predominantly shared H3K4me3/me1 occupancy but did not harbor any repressive chromatin mark (Fig. 2, D and E, and fig. S2, F and G). Most promoters losing H3K27me3 in fhPGCs between wk7 and wk9 and harbored H3K4me3 showed moderate transcriptional induction (Fig. 2F). Genes enriched in this category were associated with cell adhesion and cell signaling (Fig. 2G). In line with the reduced H3K27me3 levels, we detected elevated expression of the X chromosome–encoded H3K27me3 demethylase *KDM6A* in fhPGCs (Fig. 2H). Consequently, differential KDM6A and H3K27me3 levels may contribute to the establishment of sex-specific signaling environments in hPGCs.

Overall, the proportion of autosomal promoters occupied by repressive chromatin marks was higher in mhPGCs (45.5%) than in fhPGCs (34.9%) due to elevated H3K27me3 and H2aK119ub levels. However, in both sexes, the same combinations of repressive marks dominated at promoters, suggesting similar epigenetic gene regulatory mechanisms (fig. S2H).

### Integrative epigenetic states of promoters in hypomethylated fhPGCs

As fhPGCs exhibited lower H3K27me3 and H2aK119ub levels compared to mhPGCs, we focused on fhPGCs to study how reset hPGCs repress undesired gene expression (Fig. 2). To this end, we used an integrative self-organizing map (SOM) approach, incorporating our ChIP-seq profiles of repressive (H3K9me3, H3K27me3, and H2aK119ub), active (H3K4me3 and H3K27ac), and neutral (H3K4me1) histone modifications; ATAC-seq; published RNA-seq; and whole-genome bisulfite sequencing (BS-seq) data (*2*) generated from fGSCs and fhPGCs, which clustered 66,495 human promoters into 100 nodes (fig. S3, A and B). Repressive epigenetic marks drove the clustering of promoters into two broad groups occupied by H3K9me3 and 5mC or H3K27me3 and H2aK119ub. In fhPGCs, H3K9me3 and H3K27me3 were found at a comparatively large number of promoters, while far fewer were occupied by 5mC. Relative to fGSCs, promoter levels of all analyzed repressive histone modifications were reduced in fhPGCs. However, this was only in a fraction of promoters accompanied by an increase in active histone modifications (H3K4me3 and H3K27ac) or transcriptional activity (fig. S3, A and B).

**Fig. 3.**
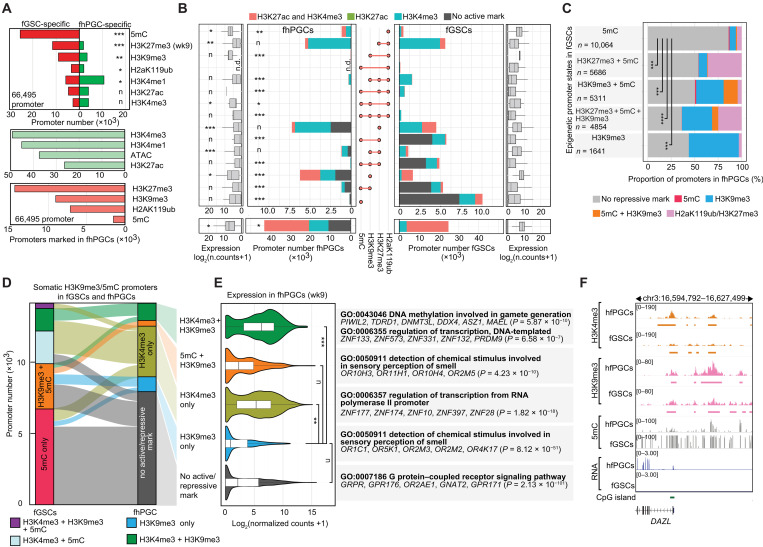
Epigenetic promoter regulation in hPGCs. (**A**) Number of promoters fGSC- (red) or fhPGC-specifically (green) occupied by the indicated modifications (top). Number of promoters occupied by the indicated epigenetic marks or overlapping with an ATAC-seq peak in fhPGCs (bottom). Chi-square goodness-of-fit effect size is depicted. (**B**) Number (inner panels) and expression level (outer panels) of promoters occupied by the indicated combinations of repressive marks. Color code indicates the co-occupancy with H3K4me3 (opal), H3K27ac (green), both marks (red), or none (gray) in fhPGCs (left) and fGSCs (right). Chi-square goodness-of-fit effect size is depicted. H3K27me3 occupancy was determined in wk9 fhPGCs/fGSCs. n.d., not detected. NC, normalized counts. (**C**) Comparison of repressive chromatin signatures at promoters in fhPGCs and fGSCs. Promoters were grouped by their repressive chromatin signatures in fGSCs, and repressive modifications in fhPGCs were quantified in each group. Chi-square goodness-of-fit test, ***P < 0.005 and ****P < 0.001. (**D**) Epigenetic states of promoters in fhPGCs and fGSCs. Depicted promoters were occupied by 5mC, H3K9me3, or both marks in the presence or absence of H3K4me3 in fGSCs. (**E**) Expression of promoter groups occupied by the indicated epigenetic modifications in wk8/9 fhPGCs. Promoter groups were defined by the fhPGC and fGSC comparison in Fig. 3D. Wilcoxon effect size is depicted (left). Enriched gene ontology terms within depicted promoter groups are shown (right). (**F**) Genome browser view of the *DAZL* locus showing the indicated epigenetic modifications in fhPGCs and fGSCs. Effect size levels as in Fig. 1.

To corroborate the findings from the SOM clustering, we analyzed epigenetic modifications at individual promoters and quantified fhPGC- and fGSC-specific and shared occupancy across both cell types (Fig. 3A). Despite its lower genomic levels in fhPGCs, H3K27me3 remained the most common repressive epigenetic mark at promoters followed by H3K9me3. While the portion of promoters occupied by repressive marks was overall substantially lower in fhPGCs (34.9%) than in fGSCs (62.8%), a small number of promoters exhibited fhPGC-specific H3K27me3, H2aK119ub, or H3K9me3 occupancy.

In fGSCs, many lowly active and inactive promoters were occupied by combinations of repressive marks including 5mC (Fig. 3B). In contrast, the most common repressive promoter signatures in fhPGCs were H3K27me3 alone, H3K9me3 alone, and H3K27me3 in combination with H2aK119ub, while only a small subset of promoters retained 5mC in combination with H3K9me3. Grouping promoters by their repressive modifications in fGSCs revealed distinct propensities for carrying a repressive epigenetic signature in fhPGCs (Fig. 3C and fig. S3C). About 86.1% of promoters marked in fGSCs by 5mC alone did not harbor any analyzed repressive epigenetic mark in fhPGCs, while this portion was only 50.2% in the case of promoters co-occupied by H3K9me3 and 5mC promoters in fGSCs, suggesting that specific repressive signatures could be more prone to be lost during the resetting of hPGCs than others.

Promoters occupied by H3K27me3 alone or in combination with H2aK119ub in fhPGCs controlled genes associated with various differentiation processes of somatic cells (fig. S3B). While these promoters showed on average low transcriptional activity in fhPGCs, most of them were co-occupied by H3K4me3 (Fig. 3B), indicative of a transcriptionally poised bivalent state observed in other cell types previously (*31*). Co-occupancy with active epigenetic marks was observed for all repressive promoter signatures, albeit with different frequencies. While virtually all H2aK119ub and H3K27me3 and 69.1% of H3K9me3 occupied promoters were bivalent, only 33.7% of promoters associated with 5mC and H3K9me3 exhibited co-occupancy with an active histone modification in fhPGCs (Fig. 3B).

In the case of H3K9me3, bivalent promoters in fhPGCs showed enrichment for transcriptionally active genes functioning in hPGC differentiation, e.g., *PIWIL1*, *DDX4*, *DAZL* (*32*), or *MAEL* (*33*), while H3K9me3 promoters lacking active chromatin marks were found at repressed ORGs (Fig. 3, D and E). Many bivalent H3K9me3 promoters, such as *DAZL*, *PIWIL1*, and *DDX4*, belonged to the previously identified group of methylation-sensitive genes (MSGs), which exhibit hPGC-specific demethylation and expression (*2*). In murine PGCs, similar promoters were suggested to be regulated by both 5mC and H2aK119ub (*8*). In line with their transcriptional activation, most of the 630 autosomal MSG promoters bore active chromatin marks and lost 5mC in wk7 fhPGCs and mhPGCs (fig. S3D). Several MSG promoters, including *DAZL*, retained H3K9me3 in fhPGCs and mhPGCs, while others exhibited H3K27me3 occupancy, specifically in mhPGCs (Fig. 3F). Besides, only a small subpopulation of 16 and 19 MSG promoters was associated with H2aK119ub in fhPGCs and mhPGCs, respectively, suggesting that coregulation of MSG promoters through PRC1 and 5mC was not typical at this stage of hPGC development (fig. S3D).

In summary, fewer promoters carried repressive modifications in fhPGCs than in fGSCs, without an overall reciprocal increase of transcription or active chromatin marks. The distribution of H3K9me3 in fhPGCs suggests that this mark mediated stable repression of specific gene groups, e.g., ORGs, and regulated transcription of germ line-associated genes like *DAZL*.

### Epigenetic compensation for the loss of 5mC in the germ line

**Fig. 4.**
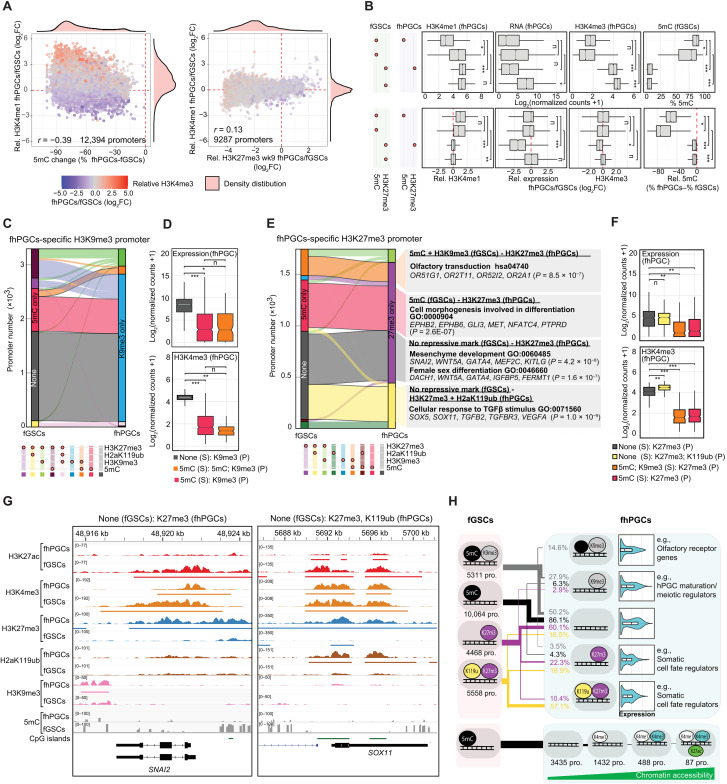
Epigenetic compensation for 5mC loss in the germ line. (**A**) Correlation between differential promoter DNA methylation and H3K4me1 levels (left) or differential H3K27me3 and H3K4me1 levels (right) in fhPGCs and fGSCs. Color code indicates differential H3K4me3 levels between fhPGCs and fGSCs. Pearson correlation coefficient is shown. Promoters co-occupied by H3K27me3 and 5mC were excluded. (**B**) Absolute (top) and relative levels (fhPGCs/fGSCs; bottom) of expression and occupancy with indicated epigenetic marks at promoters harboring the indicated epigenetic modifications in fGSCs and fhPGCs. Promoters co-occupied by H3K27me3 and 5mC in fhPGCs or fGSCs were excluded. Wilcoxon effect size is depicted. (**C**) Repressive epigenetic marks in fhPGCs and fGSCs at promoters with fhPGCs-specific H3K9me3 occupancy. (**D**) Expression and H3K4me3 levels of fhPGCs-specific H3K9me3 promoters in fhPGCs. Color code indicates epigenetic modifications in fhPGCs (P) and fGSCs (S). Wilcoxon effect size is depicted. (**E**) Repressive epigenetic states of fhPGCs-specific H3K27me3 promoters in wk9 fhPGCs and fGSCs (left). GO terms enriched within depicted promoter groups (right). (**F**) Expression and H3K4me3 levels of fhPGCs-specific H3K27me3 promoters in fhPGCs. Color code indicates epigenetic modifications in fhPGCs (P) and fGSCs (S). Wilcoxon effect size is depicted. (**G**) Genome browser view of the *SNAI2* and *SOX11* locus showing the indicated epigenetic modifications in fhPGCs and fGSCs. (**H**) Top: Comparison of repressive epigenetic promoter states in fGSCs and fhPGCs (black = 5mC; gray = H3K9me3; violet = H3K27me3; yellow = H2aK119ub). Most prominent repressive promoter states in fGSCs and the corresponding epigenetic promoter states and their expression in fhPGCs are shown. Bottom: Epigenetic states of hypomethylated promotes in fhPGCs, harboring no repressive modification (black = 5mC; white = H3K4me1; opal = H3K4me3; green = H3K27ac). Effect size levels as in Fig. 1.

To identify potential mechanisms compensating for the extensive DNA demethylation in hPGCs, we investigated promoters that exhibited reduced 5mC levels relative to fGSCs. Reduction of 5mC at promoters in fhPGCs and mhPGCs correlated with an increase in H3K4me1, which coincided only at a small number of promoters with an increase in H3K4me3, H3K27ac, or transcription (Fig. 4A and figs. S3A and S4, A and B). In contrast, promoters showing reduced H3K27me3 levels in fhPGCs were strongly occupied by H3K4me1 in both fGSCs and fhPGCs, indicating antagonism between 5mC and H3K4me1 (Fig. 4B).

Promoters occupied by repressive histone modifications, specifically in fhPGCs relative to fGSCs, fell broadly into two classes. First, promoters showing “de novo” occupancy of a repressive mark in fhPGCs while lacking repressive chromatin features in fGSCs. Second, promoters occupied by different repressive marks in fhPGCs and fGSCs indicating cell type–specific modes of gene repression. Of the 3,396 fhPGC-specific H3K9me3 promoters, 49.1% showed de novo occupancy, while 39.6% shifted from 5mC in fGSCs to H3K9me3 in fhPGCs (Fig. 4C). “De novo” H3K9me3 promoters in fhPGCs were associated with higher H3K4me3 levels and transcriptional activity than other H3K9me3-occupied promoter groups (Fig. 4D and fig. S4C), indicating potential H3K9me3-mediated modulation of gene expression rather than comprehensive transcriptional repression (*34*). Notably, promoters shifting from 5mC in somatic cells toward H3K9me3 in fhPGCs were enriched for repressed ORGs, supporting a compensatory role of H3K9me3 (fig. S4D).

Promoters de novo occupied by H3K27me3 in fhPGCs showed higher expression and H3K4me3 levels than H3K27me3 promoters occupied by other repressive chromatin marks in somatic cells (Fig. 4, E and F, and fig. S4, E and F). In all cases, fhPGC-specific H3K27me3 promoter occupancy was correlated with reduced transcription relative to fGSCs suggesting that H3K27me3 reduced or prevented gene expression (fig. S4F). Promoters gaining H3K27me3 de novo in fhPGCs were enriched for genes functioning in mesenchyme development, female sex differentiation of GSCs, migration regulation, and the cellular response to transforming growth factor–β (Fig. 4, E and G, and fig. S4G). On the other hand, promoters that switched from 5mC occupancy in fGSCs to H3K27me3 in fhPGCs showed enrichment for genes involved in morphogenesis regulation and olfactory signal transduction (fig. S4G).

Our findings suggest that distinct repressive promoter signatures have different propensities to be maintained in fhPGCs relative to fGSCs, with 5mC and H3K27me3 being most prone to be lost (Fig. 4H). Compensation for the lower 5mC levels in hPGCs by H3K9me3 or H3K27me3 only affects a small percentage of promoters. However, reduced promoter methylation correlates with H3K4me1 acquisition, while an increase in active histone marks, chromatin accessibility, or transcriptional activity is rare.

### Epigenetic regulation of TEs in hPGCs

**Fig. 5.**
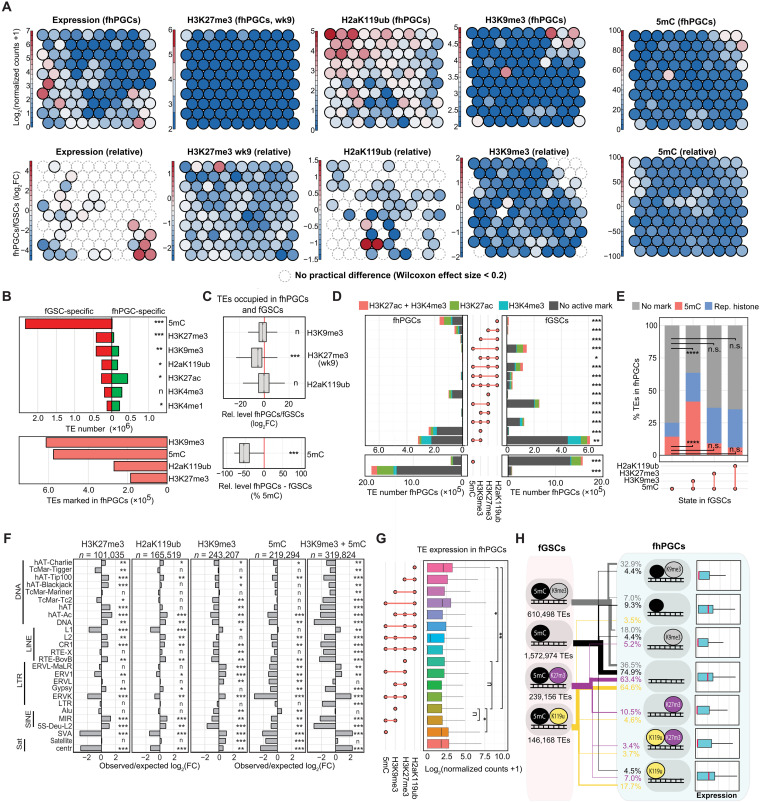
Combinatorial function of epigenetic marks in TE regulation in fhPGCs. (**A**) SOM analysis depicting clustering of 5mC, H3K9me3, H3K27me3, and H2aK119ub-marked TEs in fhPGCs (top) and relative changes between fhPGCs and fGSCs (bottom). (**B**) Number of fhPGC- (green) and fGSC-specifically (red) occupied TEs with the indicated epigenetic modifications (top). Number of TEs occupied by the indicated modifications in fhPGCs (bottom). Chi-square goodness-of-fit effect size is depicted. (**C**) Relative levels of the indicated epigenetic modifications in fhPGCs and fGSCs at TEs marked in both cell types. Wilcoxon effect size is depicted. (**D**) Number of TEs occupied by the indicated combinations of repressive marks in fhPGCs and fGSCs. Co-occupancy with H3K4me3 (opal), H3K27ac (green), both marks (red), or none of those (gray) is indicated. H3K27me3 occupancy was determined in wk9 fhPGCs. Chi-square goodness-of-fit effect size is depicted. (**E**) Proportion of TEs retaining 5mC in fhPGCs in TE subgroups occupied by the indicated combinations of repressive chromatin marks in fGSCs. Chi-square goodness-of-fit test, n.s. *P* > 0.05, ****P < 0.001. (**F**) Enrichment of TE families marked by H3K27me3, H2aK119ub, H3K9me3, and 5mC alone or in combination with H3K9me3. Chi-square goodness-of-fit effect size is depicted. (**G**) Expression level of TE subgroups occupied by the indicated combinations of epigenetic marks in fhPGCs. Wilcoxon effect size is depicted. (**H**) Repressive epigenetic states of TEs in fGSCs and fhPGCs [black = 5mC; gray = H3K9me3; violet = H3K27me3 (wk9); yellow = H2aK119ub]. Most prominent repressive states of TEs in fGSCs and the corresponding epigenetic state and expression in fhPGCs are depicted (mean of expression is indicated in red). Effect size levels as in Fig. 1.

Regulation of TEs is critical in the globally demethylated “immortal” germ line as TE expression has been previously linked to mutagenesis (*35*, *36*) and disease (*21*). Focusing on the 3,040,811 TEs covered by previously published BS-seq data (*2*), we conducted an integrative SOM clustering analysis. Analogous to the promoter SOM (fig. S3A), TEs clustered into two broad groups by their occupancy with H3K27me3/H2aK119ub and H3K9me3/5mC, respectively (Fig. 5A). Levels of repressive marks were substantially lower in fhPGCs relative to fGSCs, with 5mC and H3K9me3 being the most prominent at TEs in fhPGCs. Compared to wk7 fhPGCs or wk9 fGSCs, the number of TEs occupied with H3K27me3 was reduced in wk9 fhPGCs, while H2aK119ub occupancy appeared more stable (Fig. 5A and fig. S5, A and B). Similar to genic promoters, H3K4me1 levels increased at some TEs, showing reduced 5mC levels in fhPGCs (fig. S5C). However, SOM clustering indicated a stronger correlation between H3K4me3 and H3K4me1 levels at TEs than promoters (fig. S5D).

To obtain a more detailed view of the epigenetic environment at TEs in fhPGCs, we categorized TEs based on their epigenetic marks into fhPGC specifically, fGSC specifically, and commonly occupied (fig. S5E). In line with the above SOM analysis, more TEs were occupied by active histone modifications (H3K27ac and H3K4me3) or H3K4me1 in fhPGCs, with a pronounced decrease in the repressive 5mC and H3K27me3 modifications (Fig. 5B). Consequently, the total TE number occupied by repressive chromatin marks in fhPGCs (38.6%) was substantially lower than in fGSCs (97.2%). Even when detected at TEs in both cell types, H3K27me3 and 5mC levels were substantially lower in fhPGCs (Fig. 5C). However, 370,102 TEs exhibited fhPGC-specific occupancy of H3K9me3, H2aK119ub, or H3K27me3, indicating a potential partial compensation for the loss of 5mC (fig. S5, F and G).

In fhPGCs, H3K9me3 in combination with 5mC (10.5%), H3K9me3 alone (8.0%), 5mC alone (7.3%), H2aK119ub alone (5.4%), and H3K27me3 alone (3.3%) was the most frequent repressive signatures (Fig. 5D). Within these repressive signatures, a substantial number of TEs was co-occupied by active epigenetic marks, which was most prominent for H2aK119ub-occupied TEs. In contrast to promoters, co-occupancy of H3K27me3 and H2aK119ub at TEs was limited, potentially indicating distinct PRC1 complexes functioning at TEs and genic promoters. The epigenetic signatures detected at TEs in mhPGCs and fhPGCs were broadly similar, with the number of TEs occupied by repressive chromatin marks being higher in mhPGCs due to the wider distribution of H2aK119ub and H3K27me3 (Fig. 1E and fig. S5, H and I).

Notably, 41.3% of TEs co-occupied by 5mC and H3K9me3 in somatic cells were also occupied by 5mC in fhPGCs. This proportion was significantly smaller for TEs occupied by 5mC alone (14.3%), H3K27me3 and 5mC (9.2%), and H2aK119ub1 and 5mC (6%) in fGSCs. Overall, this suggests that 5mC-H3K9me3–coregulated TEs were particularly prone to retain a repressive state in the germ line (Fig. 5E). Concurrently, TEs co-occupied with H3K9me3 and 5mC exhibited higher levels of these marks compared with those with either mark alone, suggesting that interconnection of epigenetic pathways promoted reinforcement of the heterochromatic identity of specific genomic loci (fig. S5J). TEs occupied by H3K9me3 or H3K9me3 and 5mC were enriched for TE families containing evolutionarily young elements such as L1, SVA, or ERVK capable of independent transcription. In contrast, TEs occupied by H2aK119ub or H3K27me3 were depleted for evolutionarily young TE families and exhibited enrichment for evolutionarily old DNA transposons and L2 elements (Fig. 5F).

Expression of TEs marked by H3K27me3 or H3K9me3 was on average lower than for TEs occupied by H2aK119ub or 5mC alone (Fig. 5G). Furthermore, TEs occupied by H3K27me3 were less frequently transcriptionally induced in fhPGCs than expected. However, none of the analyzed epigenetic signatures was associated with a bias toward elevated TE expression in fhPGCs (fig. S5J).

In summary, most TEs exhibited depletion of repressive modification in hPGCs compared to surrounding GSCs, without causing broad expression changes. Heterochromatic signatures were enriched in fhPGCs at specific TE groups including H3K9me3 and 5mC at evolutionarily young TEs and H3K27me3 and H2aK119ub at some evolutionarily older TE families (Fig. 5H).

### Interconnection between gene and proximal TE expression in fhPGCs

Approximately 18% of human transcription start sites (TSSs) overlap TEs (*37*), with TE-mediated gene regulation at chimeric transcripts (*38*) and specific epialleles (*20*). To explore the connection between TE and gene expression, we relied on correlating transcription and epigenetic features between TEs and the closest gene (±100 kb to TSS) in fhPGCs and fGSCs, as functional validation was not feasible in these primary cell types. The differential expression (DE) of TEs (142,274 DE-TEs) between fhPGCs and fGSCs was positively correlated with the DE of the proximal gene (fig. S6, A and B). Most DE-TEs were found within 40 kb around the closest genic TSS, with gene and TE expression being correlated upstream and downstream of the TSS, suggesting that the connection was not solely the consequence of intronic TEs being included in the primary genic transcript (fig. S6, C and D). Focusing on the 38,745 DE-TEs up-regulated in fhPGCs, 33,030 TEs were located in the proximity of genes (±100 kb to TSS), with most lacking active chromatin features or being located downstream of a genic TSS, suggesting likely passive transcription through genic promoters (fig. S6E). Conversely, DE-TE upstream of the TSS, with active chromatin marks in fhPGCs or harboring an ATAC-seq signal, might be involved in gene regulation. These putative gene regulatory TEs were located in the vicinity of several genes mediating hPGC development, for example, *DAZL*, *PRDM1*, and *PRDM14* sharing epigenetic and transcriptional characteristics with the genic promoters (fig. S6, F to H).

### Coregulation of H3K27me3/H2aK119ub occupied TEs and promoters

Between wk7 and wk9, more than 18,000 promoters lost H3K27me3 in fhPGCs, with only a fraction gaining transcriptional activity or other repressive epigenetic marks (Fig. 2). With 184,726 and 269,230 predominantly evolutionarily old TEs being occupied by H3K27me3 and H2aK119ub in fhPGCs (Fig. 5), respectively, we asked whether these could be involved in gene regulation.

**Fig. 6.**
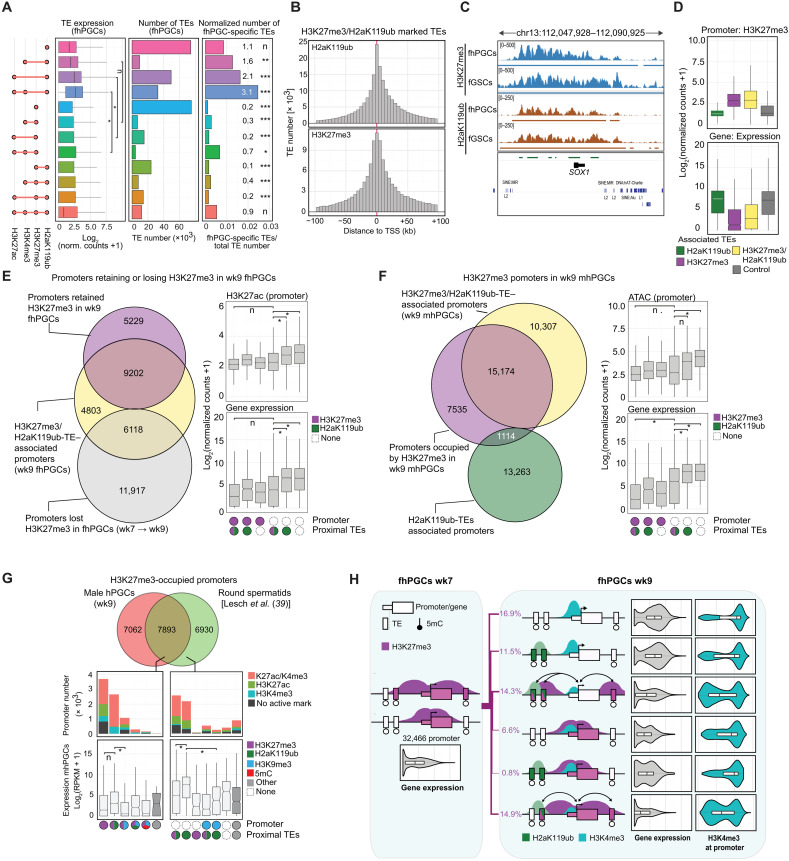
Association of H2aK119ub/H3K27me3 occupied TEs and promoters. (**A**) Expression (Wilcoxon effect size) and size of the indicated TE subgroups (left). Normalized number of fhPGC-induced TEs (Enrichment rates and chi-square goodness-of-fit effect size of fhPGC-induced TEs are shown; right). (**B**) Distribution of H3K27me3 and H2aK119ub occupied TEs in relation to the nearest genic TSS (±100 kb to TSS). (**C**) Genome browser view of the *SOX1* locus in fhPGCs and fGSCs depicting H2aK119ub and H3K27me3 occupancy. (**D**) Expression and H3K27me3 level of genic promoters associated with TEs occupied by H3K27me3, H2aK119ub, a combination of both, or neither (control) in fhPGCs. (**E**) Overlap of genic promoters that lost (gray) or retained (violet) H3K27me3 between wk7 and wk9 in fhPGCs with H3K27me3/H2aK119ub-TE–associated promoters (left). Expression and H3K27ac levels of promoters that lost (white) or retained H3K27me3 (violet) between wk7 and wk9 in fhPGCs and were associated with H3K27me3/H2aK119ub-TEs, H2aK119ub-TEs or neither (right). Wilcoxon effect size. (**F**) Overlap of genic H3K27me3-marked promoters (violet) with H3K27me3/H2aK119ub-TE (yellow) and H2aK119ub-TE–associated promoters (green) in wk9 in mhPGCs (left). Promoter expression and accessibility of the indicated promoter groups in wk9 mhPGCs (right). Wilcoxon effect size. (**G**) Overlap between promoters occupied by H3K27me3 in round spermatids [Lesch *et al.* (*39*)] and mhPGCs (top). Epigenetic state and expression in mhPGCs of promoters occupied by H3K27me3 specifically in round spermatids or both cell types (Wilcoxon effect size; bottom). (**H**) Schematic summarizing promoter activity and epigenetic state of promoter-proximal TEs. Promoters were occupied by H3K27me3 in wk7 fhPGCs and changed their epigenetic state in wk9 fhPGCs as indicated. For simplicity, promoters occupied by H2aK119ub and H3K27me3 in wk9 fhPGCs are not shown. Violin plots depict expression and H3K4me3 levels of the promoter groups in fhPGCs. Effect size levels as in Fig. 1.

While occupying overall similar TE families, H3K27me3- and H2aK119ub-occupied TEs displayed distinct characteristics in fhPGCs, with H2aK119ub-marked TEs exhibiting a higher level of co-occupancy with active chromatin marks, a substantially higher average expression, and a higher proportion of fhPGC-specific expressed TEs (Fig. 6A and fig. S7A). Concordantly, evolutionarily young SVA and ERVK elements that gained expression in fhPGCs were specifically overrepresented in the TEs co-occupied by H2aK119ub, H3K4me3, and H3K27ac but not in H3K27me3-marked TEs (fig. S7B).

Consistent with H3K27me3 and H2aK119ub domains often spanning across genes and adjacent TEs, most H3K27me3- or H2aK119ub-marked TEs were found around genic TSSs (±100 kb; Fig. 6, B and C). Next, we identified genic promoters in the vicinity (±100 kb to TSS) of H3K27me3- and H2aK119ub-marked TEs and grouped them into (i) H3K27me3-TE–, (ii) H3K27me3/H2aK119ub-TE–, and (iii) H2aK119ub-TE–associated promoters (fig. S7C). Genic promoters associated with H3K27me3-TEs or H3K27me3/H2aK119ub-TEs showed higher H3K27me3 levels and lower transcriptional activity than H2aK119ub-TE–associated promoters (Fig. 6D and fig. S7D). Genes associated with H3K27me3/H2aK119ub-TEs were enriched for functions in organism development and cellular differentiation, while genes in the vicinity of H2aK119ub-TEs were implicated in metabolic processes (fig. S7E). Moreover, most H3K27me3/H2aK119ub-TEs were occupied by H3K27me3 in fGSCs and fhPGCs, suggesting a common regulatory mechanism in both cell types, while most H2aK119ub-TEs carried this modification specifically in fhPGCs (fig. S7F).

Focusing on H3K27me3 promoters between wk7 and wk9 in fhPGCs, we found H3K27me3/H2aK119ub-TEs and H2aK119ub-TEs associated with promoters that retained or lost H3K27me3 in wk9 fhPGCs (Fig. 6E and fig. S7G). Notably, the presence of H3K27me3/H2aK119ub-TEs but not H2aK119ub-TEs was associated with low promoter activity even when no repressive mark was detected at the core promoter (Fig. 6E). Similarly, promoter-proximal H3K27me3/H2aK119ub-TEs but not H2aK119ub-TEs appeared to promote gene repression in mhPGCs (Fig. 6F and fig. S7H).

To address the dynamics of H3K27me3 occupancy during germ line development, we compared mhPGCs and human round spermatids (hRSs) using published hRS ChIP-seq data (*39*). Most H3K27me3 marked promoters in mhPGCs were also occupied by this mark in hRS (Fig. 6G), while most hRS-specific H3K27me3 promoters were associated with H3K27me3/H2aK119ub-TEs or H2aK119ub-TEs in mhPGCs. Consistent with our previous observations, promoter-proximal H2aK119ub-TEs were associated with higher gene transcription than H3K27me3/H2aK119ub-TEs (Fig. 6G).

In conclusion, H2aK119ub at TEs may have different functions when present alone or in combination with H3K27me3, which correlated with transcriptional repression. Accordingly, promoter-proximal TEs might regulate promoter activity by maintaining H3K27me3 or acquiring H2aK119ub occupancy (Fig. 6H), which merits functional validation upon establishing a suitable in vitro system.

### PRC2 inhibition results in the derepression of somatic genes in fhPGCs

**Fig. 7.**
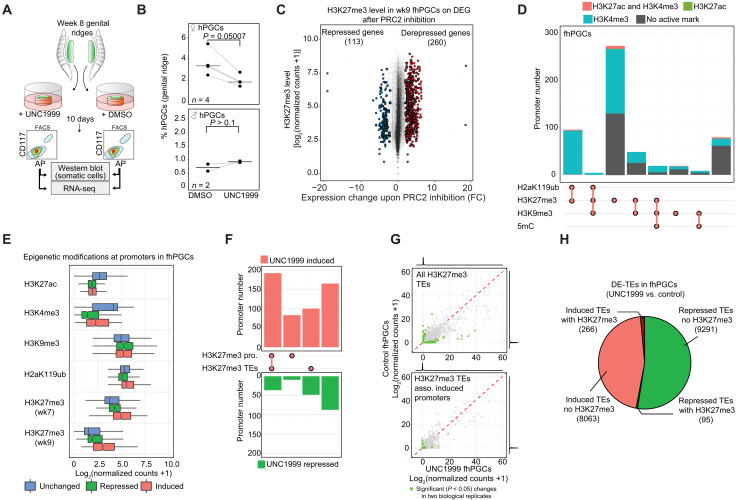
H3K27me3 is required for somatic gene repression in ex vivo cultured fhPGCs. (**A**) Schematics describing treatment of ex vivo cultured genital ridges and purification of hPGCs (see Materials and Methods). (**B**) Portion of CD117/alkaline phosphatase (AP) double-positive hPGCs within UNC1999 and control treated, ex vivo cultured female and male genital ridges. Biological replicates, *n* = 4 (fhPGCs) and *n* = 2 (mhPGCs). One-tailed, paired Student’s *t* test is shown. (**C**) H3K27me3 levels in wk9 fhPGCs at promoters associated with DE genes in control and UNC1999 inhibitor–treated fhPGCs. (**D**) Chromatin marks at promoters induced in UNC1999-treated fhPGCs. Color code indicates the co-occupancy with H3K4me3 (opal), H3K27ac (green), both marks (orange), or neither (gray). H3K27me3 levels in wk7 fhPGCs are shown. (**E**) Epigenetic modifications in fhPGCs at promoters induced (red), repressed (green), or unchanged (blue) in UNC1999-treated fhPGCs compared to control. (**F**) Portion of promoters induced (red) or repressed (green) upon UNC1999 treatment, occupied by H3K27me3 or associated with H3K27me3/H2aK119ub-TEs in wk9 fhPGCs. (**G**) DE of H3K27me3-marked TEs (wk9 fhPGCs) in UNC1999- and control-treated fhPGCs (top) and DE of H3K27me3-marked TEs associated with promoters induced upon UNC1999 treatment (bottom). Average expression of two biological replicates, with significantly (*P* < 0.05) DE-TEs depicted in green. (**H**) All DE-TEs in UNC1999- or vehicle-treated fhPGCs (*P* < 0.05, *n* = 2 biological replicates). TEs transcriptionally induced in UNC1999-treated fhPGCs with (dark red) and without (light red) H3K27me3 occupancy in wk9 fhPGCs, and TEs repressed in inhibitor-treated fhPGCs with (dark green) and without (light green) H3K27me3 occupancy.

To assess the significance of H3K27me3 for gene and TE repression in wk9 hPGCs, we used ex vivo cultures of human genital ridges in the presence of UNC1999—a specific inhibitor of the PRC2 components EZH1/EZH2 (Fig. 7A, fig. S8A, and Materials and Methods) (*40*). Pharmacologic PRC2 inhibition for 10 days reduced fhPGC numbers, which was not the case in mhPGCs (Fig. 7B and fig. S8B). A relatively rapid H3K27me3 depletion in fhPGCs resulting from globally lower H3K27me3 and higher KDM6A levels might be responsible for this phenotype (Fig 2H). To gain further insight into this phenotype, we examined the transcriptional consequences of PRC2 inhibition in the female germ line by RNA-seq.

Consistent with the repressive function of H3K27me3, we found 260 of 373 significantly differentially expressed (DE) genes, derepressed upon inhibitor treatment relative to control (Fig. 7C). Most promoters of derepressed genes in fhPGCs and GSCs were occupied by H3K27me3 in their respective cell types (Fig. 7, D and E, and fig. S8C). However, the derepressed genes represented only a fraction of the total number of H3K27me3-occupied promoters, which might indicate that other factors, such as a lack of transcription factors (TFs), may prevent the expression of some genes (fig. S8D). The genes induced upon PRC2 inhibition and occupied by H3K27me3 in fhPGCs included those involved in somatic cell development, e.g., *GSC* or *HAND1*, which could contribute to the loss of germ line identity (fig. S8E).

While several promoters derepressed upon UNC1999 treatment were associated with H3K27me3-occupied TEs (Fig. 7F), the vast majority of TEs occupied by H3K27me3 in fhPGCs did not show expression changes (99.86%, 184,460 TEs; Fig. 7G). Of the 8329 TEs induced in fhPGCs, only 266 were occupied by H3K27me3, suggesting that H3K27me3 was not primarily required for TE repression but contributed to transcriptional regulation of genic promoters in their vicinity, possibly through enhancer repression (Fig. 7H).

### H3K9me3 is enriched at evolutionarily young TEs in fhPGCs

**Fig. 8.**
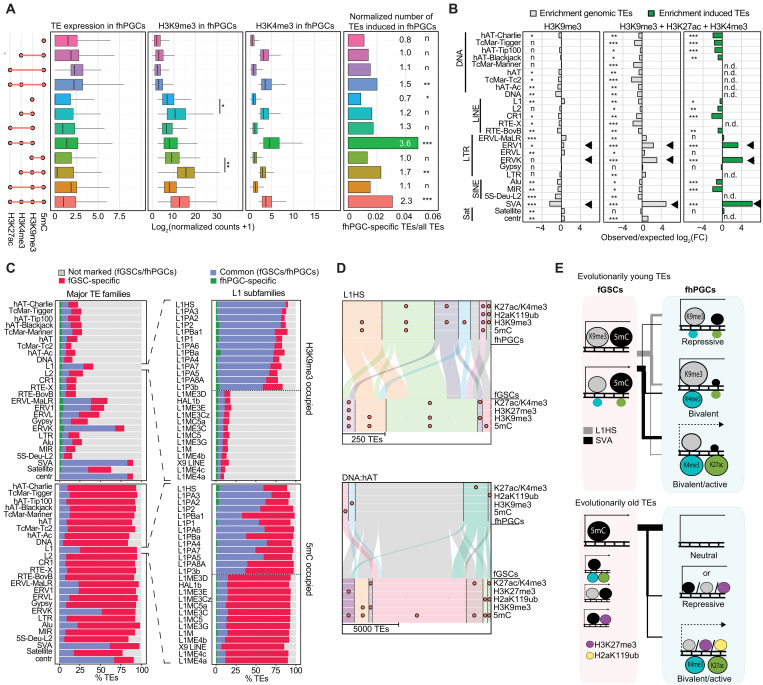
H3K9me3 is more prominent at evolutionarily young TEs than 5mC in fhPGCs. (**A**) Expression and occupancy with indicated epigenetic marks of TEs falling into the indicated TE subgroups in fhPGCs (Wilcoxon effect size). Right panel depicts number of fhPGC-induced TEs normalized to the TE subgroups size (enrichment rates and chi-square goodness-of-fit effect size of fhPGC-induced TEs are shown). (**B**) Enrichment of TE families in TE subgroups marked by H3K9me3 alone or in combination with H3K4me3 and H3K27ac. Enrichment among genomic TE loci (gray) and fhPGC-specifically induced TEs (green). Note that specific evolutionarily young TE families (arrows) are enriched in H3K9me3, H3K4me3, and H3K27ac co-occupied TEs but not in TEs occupied by H3K9me3 alone. Chi-square goodness-of-fit effect size is shown. (**C**) Portion of TEs within major TE families (left) and LINE:L1 subfamilies (right) showing H3K9me3 (top) or 5mC (bottom) occupancy specifically in fhPGCs (green), fGSCs (red), or both cell types (blue). LINE:L1 subfamilies were ranked by the frequency of H3K9me3 occupancy. Shown are LINE:L1 subfamilies representing the highest and lowest H3K9me3 occupancy (separated by dotted line), respectively. (**D**) Comparison of the epigenetic state of L1HS (top) and DNA:hAT elements (bottom) in fhPGCs and fGSCs. H3K27me3 occupancy in wk9 fhPGCs/fGSCs is shown. (**E**) Model comparing TEs occupied by 5mC and H3K9me3 in fhPGCs and fGSCs. Epigenetic states of evolutionarily young L1HS (gray bar) and SVA (black bar) elements (top) and evolutionarily old TEs (bottom) in fhPGCs and fGSCs are shown. Effect size levels as in Fig. 1.

Despite covering fewer TEs than in fGSCs, H3K9me3 and 5mC were the most common repressive marks at TEs in fhPGCs (Fig. 5B). In the absence of active epigenetic marks, H3K9me3-labeled TEs exhibited low transcriptional activity, which was elevated in a subgroup of H3K9me3 TEs co-occupied by H3K4me3/H3K27ac (bivalent TEs) comprising evolutionarily young and transcriptionally active TE families, e.g., ERVK, SVA, or ERV1 (Fig. 8, A and B, and fig. S9, A and B). In contrast, bivalent TEs marked by 5mC in the absence of H3K9me3 were enriched for Alu and SVA elements (fig. S9B).

The epigenetic state of TEs within TE classes and families varied substantially resulting in different portions of TEs residing in a bivalent, repressive (H3K9me3/5mC/H3K27me3/H2aK119ub-occupied), active (H3K4me3/H3K27ac-occupied), or neutral states (not occupied by any mark; fig. S9C). In contrast to older TE classes such as DNA transposons, which were occupied by 5mC in fGSCs and largely devoid of detectable repressive marks in germ cells, most of evolutionarily young TEs, e.g., L1HS, ERVK, or SVA elements retained at least one repressive epigenetic mark in fhPGCs, with H3K9me3 being the most prominent among these (Fig. 8, C and D, and fig. S9C). Accordingly, most SVA and L1HS elements were occupied by H3K9me3 in fGSCs and fhPGCs, with an equal portion of elements exhibiting lower H3K9me3 levels in fhPGCs. Nevertheless, the number of elements that exhibited concurrently increased active H3K27ac and H3K4me3 marks in fhPGC was substantially larger in the case of the SVA elements, in line with the greater number of SVA loci showing increased transcription in fhPGCs (Fig. 8D and fig. S9, D and E). The highest H3K9me3 levels were found at TEs co-occupied by intermediate H3K4me3 and low H3K27ac levels, suggesting that H3K4me3 deposition might trigger a feedback loop promoting H3K9me3 accumulation (fig. S9, E to G). Notably, the correlation of differential SVA and proximal gene expression was much weaker than that for older TEs, e.g., DNA:hAT or the bulk of DE-TEs, suggesting that SVA activation in fhPGCs was, at least in part, not driven by changes in neighboring gene expression (figs. S6A and S9H). Furthermore, evolutionarily old elements, e.g., DNA:hAT specifically deprived of repressive marks in fhPGCs, did not show a reciprocal increase in active histone modifications, indicating the absence of euchromatization (Fig. 8D and fig. S9, D to F). In conclusion, the bivalent state of most evolutionarily young SVA and L1HS elements in fhPGCs suggests that repressive and activating mechanisms were antagonistically targeting these TEs simultaneously, resulting in varying degrees of euchromatisation between and within TE families with only a fraction of TEs exhibiting increased expression in fhPGCs (Fig. 8E).

### SETDB1-H3K9me3 axis regulates evolutionarily young TEs and specific germ cell marker genes in the hypomethylated genome

To explore the functional relevance of H3K9me3 for TE and gene regulation in a hypomethylated genome, we used naïve human embryonic stem cells (nhESCs) (*41*). The nhESCs serve as a surrogate system for hypomethylated hPGCs because, currently, there is no genetically manipulatable in vitro system that can faithfully recapitulate epigenetic resetting in hPGCs.

**Fig. 9.**
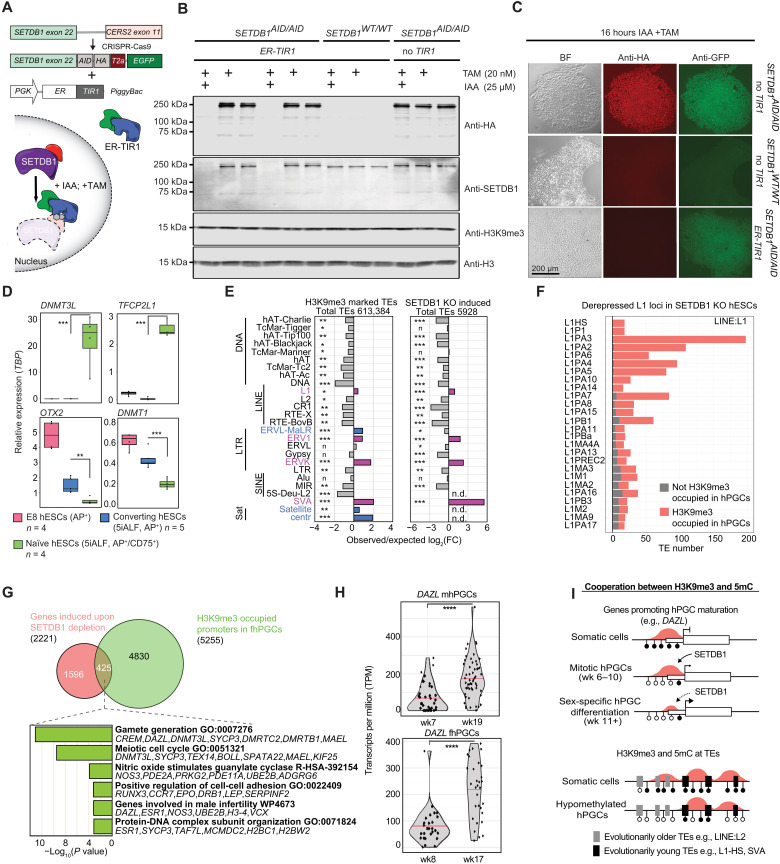
H3K9me3 is essential for TE and gene repression in the hypomethylated human genome. (**A**) Schematics of the SETDB1-AID system, established in *NANOS3-tdTomato* hESCs by introducing a *HA-AID-T2A-EGFP-tag* into the *SETDB1* locus and random integration of an estrogen receptor-coupled TIR1 (*ER-TIR1*) transgene (see Materials and Methods). (**B** and **C**) Western blot (B) and immunofluorescence staining (C) analyzing SETDB1 levels in *SETDB1-WT* and *SETDB1-AID* hESCs, with or without *ER-TIR1* transgene. Cells were cultured in Essential 8 medium and treated for 16 hours with 20 nM tamoxifen and 25 μM auxin (IAA) or vehicle (vehicle treatment only shown in B). Note that detection of EGFP but loss of SETDB1-HA-AID in *SETDB1-AID*; *ER-TIR1* hESCs due to T2A cleavage (C). (**D**) Marker gene expression in FACS-purified naïve (5iALF, AP^+^/CD75^+^), converting (5iALF, AP^+^), and Essential 8-cultured (AP^+^) *SETDB1-WT* hESCs (*n* ≥ 4 independent technical replicates). Student’s *t* test, **P < 0.01 and ***P < 0.005. (**E**) Enrichment of H3K9me3-marked TEs in fhPGCs (left) and TEs transcriptionally induced upon SETDB1 depletion in naïve hESCs (right). Enrichment under both conditions (violet) and fhPGC-specific enrichment (blue). Chi-square effect size is shown. Effect size levels as in Fig. 1. (**F**) Number of TEs belonging to LINE:L1 subfamilies induced upon SETDB1 depletion. Color code indicates H3K9me3 occupancy in fhPGCs. (**G**) Overlap between genes induced upon SETDB1 depletion and H3K9me3-occupied genes in fhPGCs (top). GO analysis of genes induced upon SETDB1 depletion and marked by H3K9me3 in fhPGCs (bottom). (**H**) *DAZL* expression in mhPGCs and fhPGCs. scRNA-seq data reanalyzed from GSE63818 (*30*). Sleuth’s likelihood ratio test: ****P < 0.001. (**I**) Model of H3K9me3-mediated gene and TE regulation in hPGCs and somatic cells. [Fig F1]

Here, we focused on the histone methyltransferase SETDB1, which deposits H3K9me3 at TEs in a KAP1-dependent manner (*15*, *22*). To efficiently deplete SETDB1 in hESCs, we modified the AID-degron system by inserting an AID-T2A–enhanced green fluorescent protein (EGFP) tag into the *SETDB1* locus (*SETDB1-AID*) and expressed a fusion protein consisting of TIR1 and the estrogen receptor (ER-TIR1; Fig. 9A). Targeting TIR1 to the nucleus promoted degradation of the nuclear protein SETDB1 after stimulation with auxin (IAA) and tamoxifen (TAM). At the same time, EGFP levels remained unaffected, indicating an efficient cleavage of the T2A peptide (Fig. 9, B and C, and fig. S10A). Depletion of SETDB1 in hESCs cultured in conventional Essential 8 medium resulted in cell death after 6 to 8 days (fig. S10B). Next, we converted conventionally cultured hESCs into the naïve state using the previously published five inhibitors (5iALF) protocol (*41*), FACS-purified the naïve cell population growing on feeder cells, and validated the expression of key naïve marker genes *DNMT3L* and *TFCP2L1* (Fig. 9D and fig. S10, C and D) as well as the genome-wide loss of 5mC (fig. S10E). Notably, parental *SETDB1-wild type* (*SETDB1-WT*); *ER-TIR1* and *SETDB1-AID*; *ER-TIR1* hESC lines grew similarly and converted with similar efficiency to the naïve state (fig. S10, B and F).

SETDB1 depletion in the naïve state for 4 days resulted in the induction of 2221 genes and 5928 TEs relative to *SETDB1-WT* controls (fig. S11, A and B). TEs induced in SETDB1-depleted nhESCs showed enrichment for TE families that tend to be occupied by H3K9me3 in fhPGCs (Fig. 9E and fig. S11C). Notably, many evolutionarily young TEs induced upon SETDB1 depletion, e.g., L1HS, SVA, LTR12C, and ERVK elements (Fig. 9F and fig. S11, D and E) were occupied by H3K9me3 in fhPGCs, suggesting that these elements are directly regulated through SETDB1 in both cell types.

Next, we identified genes likely directly regulated through SETDB1 by intersecting genes induced upon SETDB1 depletion and occupied by H3K9me3 in hPGCs. Among the direct putative SETDB1 targets are several regulators of gametogenesis, including *DAZL*, *CREM*, and *MAEL* (Fig. 9G). However, most genes induced in SETDB1-depleted nhESCs were not occupied by H3K9me3 in fhPGCs, suggesting that H3K9me3 may not directly regulate these genes in nhESCs (Fig. 9G and fig. S11F). Alternatively, H3K9me3 might regulate more genes in nhESCs than in fhPGCs. A substantial proportion of the 3697 DE genes harbored DE-TEs in their vicinity. Here, the expression of DE genes and upstream-located DE-TEs were positively correlated, supporting a link between TE and gene expression (fig. S11G). Notably, among the induced genes that harbored induced DE-TEs upstream of their promoter were *DAZL* (*32*), *EED* (*42*), and *FGF2* (*43*), which have been shown to participate in germ line development (fig. S11G).

In conclusion, H3K9me3 might play a role in the temporal control of germ line gene expression in hypomethylated hPGCs. Accordingly, in wk8/9 hPGCs when the *DAZL* locus had lost virtually all DNA methylation but remained H3K9me3-occupied, *DAZL* expression was weaker than in later germ line development (Fig. 9H and fig. S11H). In addition, H3K9me3 at many evolutionarily young TEs, including L1HS and SVA elements, was essential for their repression in the hypomethylated genome (Fig. 9I).

## DISCUSSION

Our extensive epigenomic analysis provides further insights into the comprehensive epigenetic resetting of the human germ line, comprising canonical repressive chromatin marks, with some sex-specific differences. Because heterochromatic features were maintained only in a subset of genes and TEs, transcriptional repression in hPGCs likely relies substantially on the absence of activating TFs or signaling, which requires consideration for the development of human in vitro gametogenesis (*44*).

Most genic promoters, hypomethylated in fhPGCs, lacked enrichment of canonical repressive chromatin marks such as H3K9me3 or H3K27me3 or acquisition of H3K4me3 and transcriptional activity, while many of these promoters had higher H3K4me1 levels than in GSCs. In mammalian cells, unimodally distributed H3K4me1 at promoters has been designated as a poised epigenetic state with reduced transcription activity (*45*). In zebrafish, “placeholder” nucleosomes marked by H3K4me1 occupy transcriptionally quiescent promoters in the absence of 5mC before zygotic genome activation (*46*). Consequently, H3K4me1 might establish a poised epigenetic state upon promoter demethylation in the germ line.

Previously, MSGs were identified that become specifically demethylated and expressed in the human germ line (*2*). A subpopulation of MSGs, including *DAZL*, an essential driver of PGC differentiation (*32*), retained H3K9me3 in hypomethylated hPGCs. Here, the lower *DAZL* expression in mitotically active fhPGCs compared to later fetal germ cells, along with the *DAZL* activation in hypomethylated naïve hESCs and murine ESCs (*15*) upon SETDB1 depletion, supports the notion that 5mC and H3K9me3 coregulate the transcription level and timing of some MSGs during germ cell maturation. Another gene group enriched for H3K9me3 occupation in hPGCs was ORGs, which merits consideration because the stochastic reduction of H3K9me3 levels at olfactory receptor alleles triggers the expression of a single ORG in murine olfactory sensory neurons (*47*, *48*). In mice, biases in ORG expression might be inherited transgenerationally (*49*), which could explain the need for strong repression of ORGs in the germ line.

We uncovered male-specific H3K27me3 and H2aK119ub occupancy of genes associated with cell signaling and adhesion in wk9 hPGCs, which could promote subsequent sex-specific germ cell development. The more pronounced genome-wide reduction of H3K27me3 and H2aK119ub in fhPGCs could be associated with the reactivation of Xi, which is, in part, repressed by H3K27me3 (*12*, *50*). Consistently, we detected elevated expression of the X chromosome–encoded H3K27me3 demethylase *KDM6A* in fhPGCs. The bi-allelic expression of X chromosomal genes is already detected in wk4 fhPGCs (*30*), with most fhPGCs show clear signs of two active X chromosomes by wk7 (*51*). However, complete epigenetic Xi reactivation might only be completed later.

In murine PGCs, H3K27me3 occupancy reduces in males and females between the mitotically active gonadal PGC state and the onset of sex differentiation, with male murine PGCs substantially gaining H3K9me3 (*7*). The transcriptional network driving PGC development has diverged in humans and mice, potentially resulting in different resetting kinetics (*52*). Accordingly, murine PGCs undergo profound DNA demethylation during the transition from migratory to gonadal stage (*8*), while hPGCs lose the bulk of 5mC before the gonadal stage (*2*, *53*). Consequently, the difference in histone modification levels between hPGCs and murine PGCs might reflect species-specific kinetics in epigenetic remodeling.

Our analysis also reveals that H3K27me3-occupied regions containing evolutionarily old TEs near genic promoters could participate in gene regulation in hPGCs. Notably, H3K27me3-rich regions in the human genome have recently been shown to loop to proximal or distal promoters mediating gene repression (*54*). Hence, it is conceivable that during evolution, some promoter-proximal TEs might have been co-opted to recruit TFs regulating H3K27me3 occupancy and consequently affecting proximal gene expression, for example, through enhancer inactivation, which merits functional validation in the future.

The epigenetic environment of TEs in hPGCs varied substantially among TE classes and families. Most evolutionarily old TEs were devoid of repressive or active chromatin features in the hPGCs, likely through accumulated mutations impairing promoter function during evolution. In contrast, most evolutionarily young TEs, such as SVA elements, remained repressed predominantly through H3K9me3 but gained H3K4me3, indicating that activating and repressing pathways target these loci simultaneously. Increased TE expression following SETDB1 depletion in nhESCs supports an essential role of H3K9me3 for the suppression of young TEs, which is likely mediated through Krüppel-associated box zinc finger proteins that gain expression in hPGCs and target evolutionarily young TEs (*2*, *55*). In mice, small noncoding RNAs such as piRNAs have been shown to mediate TE repression through H3K9me3 deposition, albeit in later germ cell development (*56*). While there are no data supporting an active piRNA pathway in mitotically active hPGCs, other layers of defense, such as tRNA fragments (*57*) and RNA modifications (*58*), might contribute to TE repression, H3K9me3 deposition, or prevention of transcriptional activation.

The loss of DNA methylation in mammalian PGCs is obligatory for imprint erasure and germ cell maturation. However, the evolutionary benefit of reducing levels of major repressive histone modifications in the hypomethylated germ line has yet to be explored. In mammals, mechanisms such as the mitochondria bottleneck, mPGC apoptosis, and fetal oocyte attrition, an elimination process of meiotic prophase I oogonia, suggest that the germ line can function as a filter to prevent the propagation of disadvantageous traits (*59*, *60*). Notably, the completion of the DNA demethylation in murine germ cells falls within a period of increased mPGC apoptosis (*23*, *61*). In addition, fetal oocyte attrition has been linked to elevated L1 expression levels, suggesting the existence of a selection mechanism sensing elevated TE expression (*62*). Hence, it is tantalizing to consider that reducing the repressive chromatin state in a substantial portion of the genome could promote a purifying selection against the hPGCs harboring aberrantly active TEs.

Our extensive epigenomic characterization of hypomethylated hPGCs reveals an exemplary balanced gene regulatory system relying on localized maintenance of repressive chromatin and the absence of inductive cues. These findings can serve as a basis for decoding the epigenetic resetting for successful gametogenesis and the totipotent zygote.

## MATERIALS AND METHODS

### Ethics statement

Permission to use the human embryonic tissues in this study was granted by the National Health Service Research Ethical Committee, UK [Research Ethics Committee number (REC number): 96/085] with informed patient consent. Medical or surgical termination of pregnancy was carried out at Addenbrooke’s Hospital, Cambridge, UK.

### Collection of murine gonadal cells

Animal studies were authorized by a U.K. Home Office Project License (license number PE596D1FE) and carried out in the home office–designated facility at the Wellcome Trust/Cancer Research UK Gurdon Institute, Cambridge, UK in accordance with the Institute Animal Care and Use Committee guidelines. Timed matings of ΔPE-Oct4-GFP (GOF-GFP) mice (*63*, *64*) were used to generate embryonic day (E)12.5 embryos, with noon of the day of vaginal plug detection was scored as E0.5 after coitum. Pregnant females were euthanized, the embryos were extracted from the uteri, and genital ridges were dissected in cold phosphate-buffered saline (PBS). Limb development and number of tail somites were used to stage the embryos (*65*). Genital ridges were incubated for 5 min in 0.25% Trypsin/EDTA at 37°C and dissociated by pipetting after the addition of Dulbecco’s Modified Eagle Medium: Nutrient Mixture F12(DMEM) F/12 (Gibco) and 10% fetal calf serum (FCS). After pelleting through centrifugation for 5 min at 500*g*, cells were resuspended in PBS, 3% FCS, 5% EDTA, and mGSCs and murine PGCs were separated by FACS using GFP marking the germ cells. Cells were washed once with PBS and stored on −80°C. The embryos sex was determined by polymerase chain reaction (PCR) as described previously (*66*).

### Collection of human gonadal cells

Purification of hPGCs and hGSCs was performed as described before (*28*). In brief, embryonic crown-rump length and anatomical features and approximate pregnancy length were used to determine the developmental stage of human embryos with reference to Carnegie staging (CS), while the embryo sex was determined by PCR (*66*). Human genital ridges were dissected from male and female wk7 to wk10 embryos and dissociated with collagenase IV (Sigma-Aldrich, C5138) and deoxyribonuclease I (DNase I) in DMEM F/12 (Gibco) at 37°C and resuspended in PBS, 3% FCS, and 5% EDTA. To efficiently separate hPGCs from GSCs, gonadal cells were incubated with Alexa Fluor 488–conjugated anti-alkaline phosphatase (5 μl; BD Pharmingen 561495) and allophycocyanin(APC)-conjugated anti–c-KIT (5 μl; Invitrogen CD11705) antibodies for 15 min at room temperature (RT) in an orbital rotor (10 rpm) in the dark. After washing and filtering through a 35-μm cell strainer, the cells were FACS-purified using SH800Z Cell Sorter (Sony). To validate the purity of the alkaline phosphatase and cKIT double-positive and double-negative cell populations, about 100 cells were sorted onto poly-l-lysine slides (Thermo Fisher Scientific), fixed in 4% paraformaldehyde (PFA), and subsequently subjected to alkaline phosphatase staining performed with a leukocyte alkaline phosphatase kit (Sigma-Aldrich). Only samples with >97% purity were used for epigenomic analysis. Cells for ChIP-seq analysis were stored in 20 μl of Nuclei EZ storage buffer (Nuclei EZ prep nuclei isolation kit, Sigma-Aldrich) at −80°C, and cells for Western blot were stored in 10 μl of PBS at −80°C.

### Generation of ChIP-seq libraries

Overall, the histone modification ULI-NChIP-seq was conducted as described previously (*26*, *28*), while a *Drosophila* S2 cell spike-in was introduced, allowing the adjustment for genome-wide changes of histone modification levels. The spike-in was generated by FACS (SH800Z Cell Sorter, Sony) sorting S2 cells into 10,000 cell aliquots and stored in 20 μl of Nuclei EZ storage buffer at −80°C. Before conducting the ChIP, FACS-purified hPGCs, GSCs, and S2 cell spike-in were thawed on ice and S2 cells corresponding to 1/20th of the hPGC and GSC number were added to the hPGC and GSC samples, respectively. One aliquot of spike-in was used for one set of ChIPs analyzing one histone modification. ChIPs on all biological samples used to analyze one histone modification were conducted in parallel. Samples including the spike-in were digested with MNase (NEB) and subsequently incubated with 11 μl of 100 mM EDTA to terminate MNase activity. Per IP sample, 5 μl of protein A Dynabeads with 5 μl of protein G Dynabeads was combined and blocked by incubation with 500 μl of blocking buffer [yeast tRNA (100 μg/ml) and 0.1% bovine serum albumin (BSA) in IP buffer] for 1.5 hours while rotating at 4°C. The cell lysate was precleared for 2 hours through incubation in the presence of 5 μl of blocked protein A/G beads. In parallel, the antibody-bead complex was generated by incubating per IP 5 μl of blocked protein A/G beads with specific antibodies (table S1) for 3 hours while rotating at 4°C. After the removal of the protein A/G beads, the precleared cell lysate was combined with the antibody-bead complex and incubated for 16 hours rotating at 4°C. Unbound chromatin was removed, and beads were washed consecutively for 4 min for (i) two times with low salt wash buffer, (ii) two times with high salt buffer, and (iii) two times with LiCl wash buffer [20 mM tris-HCl (pH 8.0), 2 mM EDTA, 250 mM LiCl, 1% NP-40, and 1% sodium deoxycholate]. The antigene-bound DNA was eluted from the beads through incubation in Proteinase K digestion buffer [20 mM Hepes (pH 8.0), 1 mM EDTA, 0.5% SDS, ribonuclease (1 mg/ml), and Proteinase K (0.4 mg/ml)] for 15 min at 55°C and 1 hour at 65°C. The eluted DNA was purified using AMPure XP beads (Beckman Coulter), eluted in 20 μl of EB buffer (MinElute Reaction Cleanup Kit, Qiagen), and subsequently used to generate libraries for next-generation sequencing using the KAPA Hyper Prep Kit (KAPA Biosystems). To minimize adaptor dimer formation, the NEBNext Adaptor and NEBNext Index PCR Primers from the NEBNext Multiplex Oligos for Illumina (NEB, E7335S) were used. ChIP-seq libraries were purified by AMPure XP beads with double-sided size selection and analyzed on 2200 TapeStation 2200 (Agilent) to verify the absence of adaptor dimers. After quantification and quality validation, libraries were subjected to paired-end sequencing on HiSeq 4000 sequencing system (Illumina), resulting in 27 to 73 million paired-end reads per sample. The sensitivity and specificity of all antibodies used in this study were extensively tested by the manufacturer and our laboratory by ChIP–quantitative PCR (qPCR) and ULI-NChIP-seq. Notably, the H3K27me3, H3K4me3, H3K27ac, and H3K4me1 antibodies used here are identical to the antibodies used in a previous study from our laboratory (*28*).

### Generation of RNA-seq libraries

Between 1000 and 5000 cells were FACS-sorted directly into the extraction buffer (PicoPure RNA Isolation Kit, Applied Biosystems), snap frozen, and stored at −80°C. RNA was extracted using the PicoPure RNA Isolation Kit including an on-column DNase I digest (Qiagen, 79254) following the manufacturer’s instructions. RNA-seq libraries were generated from 2 to 5 ng of total RNA using the NEBNext Single Cell/Low Input RNA Library Prep Kit for Illumina (NEB, E6420) and NEBNext Multiplex Oligos for Illumina (NEB, E7335S). Library quality was accessed by TapeStation 2200 (Agilent) with High Sensitivity D1000 ScreenTape (Agilent), quantified, and multiplexed. The multiplexed libraries were subjected to paired-end 100–base pair (bp) sequencing on HiSeq 4000 sequencing system (Illumina), resulting in >30 million reads per sample.

### Ex vivo culture of human genital ridges

The ex vivo culture of human genital ridges was adapted from previously published protocols (*67*–*69*). The CS stage of the embryos was determined through crown-rump length and anatomical features, and the embryo sex was determined by PCR (*66*). Human genital ridges were dissected from wk8 to wk9 embryos and cut sagittally to generate two to three fragments per genital ridge to allow better penetration of inhibitors. The fragments were placed on agar blocks [1% agar and minimum essential medium–α (MEM-α) (22561021, Gibco)], and culture medium [10% FCS, penicillin-streptomycin (100 U/ml; 15140122, Gibco), and MEM-α (Gibco)] was added until the edge of the agar block to generate an air-liquid interface. The fragments originating from one genital ridge were cultured for 10 days in the presence of 1 μM UNC1999 [in dimethyl sulfoxide (DMSO) (CAY14621, Cambridge Bioscience)], while fragments of the second genital ridge of the same embryo were cultured in the presence of DMSO. Subsequently, hPGCs and GSCs were FACS-purified as described in the “Collection of human gonadal cells” section. A portion of GSCs and all hPGCs were sorted directly into extraction buffer (PicoPure RNA Isolation Kit, Applied Biosystems) and used for RNA-seq, while some somatic cells were used to verify the reduction of H3K27me3 levels by Western blot.

### Chromatin ChIP-seq data processing

Adapter-and quality-trimmed reads were mapped to the human [University of California, Santa Cruz (UCSC) GRCh38/hg38)] and *Drosophila* (UCSC dm6) reference genomes. Using bowtie (http://bowtie-bio.sourceforge.net; version: 1.1.0) with parameters “-m1 –v2 –best –strata” reads were selected that uniquely align to single human genomic repeat copies (3,040,811 TEs, RepeatMasker) or promoters [−2000, +500 bp TSS, 66,495 promoters, Genecode promoters (version 31)], allowing two mismatches. A spike-in normalization factor was determined across all IP samples generated with the same antibody, through the total number of reads specifically mapping *Drosophila* genome. IP read counts were normalized to input reads, the length of the TE or promoter, and spike-in normalization factor. Genome-wide profiles were generated by applying the spike-in normalization factor to sliding 50-nt windows (25-nt offset). Peaks for ChIP-seq libraries were called following the ENCODE replicated peak calling guidelines (www.encodeproject.org/pipelines/ENCPL272XAE/) (*70*) with modifications to accommodate for paired-end libraries. Peaks were initially called for each biological replicate, for the pooled replicates, and for the pooled pseudoreplicates of each biological replicate using MACS2 (2.1.2) with a relaxed *P* value threshold of 0.05. Each pseudoreplicate consists of half the reads of each biological replicate, chosen at random without replacement. Broad peaks from the pooled replicate set were retained if they overlapped peaks from both biological replicates or peaks from both pooled pseudoreplicates. This peak calling strategy allows for the retention of marginal peaks in one replicate to be rescued by a strong biological replicate. To obtain a final high confidence peak set, the reproducible peaks were further filtered using the MACS2 *q* value (false discovery rate < 0.0001 for ATAC peaks and false discovery rate < 0.001 for histone peaks). Promoters or TEs were considered as occupied by a specific histone modification when overlap with a broad peak was detected, and the normalized number of reads mapping to these regions exceeded the average number of normalized reads in negative regions.

### SOM analysis

SOMs for TEs (3,040,811 TEs, RepeatMasker) and promoters [66,495 promoters, Gencode promoters (version 31)] were generated with R kohonen package (*71*) using 10 × 10 circular grids. Datasets included in the SOM analysis comprised all normalized ChIP-seq datasets on fhPGCs and fGSCs (spike-in, input, and size-normalized), ATAC-seq data on fhPGCs (male ATAC-seq data, GSE159654), RNA-seq data [National Center for Biotechnology Information (NCBI) Sequence Read Archive (SRA): SRP057098], and whole-genome BS-seq (NCBI SRA: SRP057098). Reads on TEs and promoters were quantified with the R Bioconductor edgeR package. ATAC-seq and RNA-seq datasets were normalized by the total number of reads, and ChIP-seq reads were normalized by spike-ins. ChIP-seq, ATAC-seq, and RNA-seq datasets were further normalized to the length of TE or promoter regions. BS-seq datasets were analyzed as described (*2*), and DNA methylation levels of TEs and promoters were quantified as average over all CpGs with more than 5× BS-seq read coverage. ChIP-seq, ATAC-seq, and RNA-seq datasets were subsequently log_2_-transformed, and all datasets were scaled. SOMs were trained for 5000 cycles (rlen = 5000) with linearly declining learning rates (alpha = 0.09, 0.01).

### TE expression analysis

Analysis of TE expression in hPGCs and GSCs (using previously published RNA-seq data, NCBI SRA: SRP057098) and in ex vivo cultured fhPGCs and fGSCs was conducted as previously described (*2*). To evaluate single TE copies (3,040,811 TEs, RepeatMasker), adapter-trimmed RNA-seq reads were mapped to the human reference genome (GRCh38/hg38) with bowtie (parameters: -m1 –v2 –best –strata), allowing two mismatches and keeping uniquely mapping reads only. Reads aligned to repeat regions were quantified by featureCounts, normalized by the total number of RNA-seq reads that mapped to protein-coding gene regions, and normalized to the repeat length. The R Bioconductor edgeR package was used to determine differential repeat expression across samples. Because total repeat expression was underestimated by rejecting multiply mapping reads, RNA-seq reads were further mapped with bowtie2 using default parameters to evaluate the average expression of repeat families. In addition, repeats that intersected with exons of annotated long noncoding RNAs (lncRNAs) were evaluated independently by using TopHat2 and considering the total read counts for the annotated lncRNA transcripts. Differentially TE expression between hPGCs and GSCs was defined as (i) ≥4-fold difference of expression and (ii) the higher expressed TEs log_2_(RPKM + 1) > 0.4.

To determine the differential TE expression in *SETDB1-WT* and *SETDB1-AID* hESCs, adapter-trimmed RNA-seq reads were mapped to a reference set of human TEs set using SQUiRE (*72*) package. Mapped reads were quantified by SQUiRE Call, which incorporated the R Bioconductor package DESeq2 (*72*).

### Categorizing TE and gene expression

Thresholds for categorizing TE and gene expression (Fig. 1) in hPGCs and GSCs into “not expressed,” “lowly expressed,” and “expressed” were empirically set on the basis of the bimodal distributed expression in hPGCs. The mode around zero was designated as not expressed, with the second mode representing expressed genes. The region between these two modes was designated as lowly expressed. Thresholds for TE expression [log_2_(RPKM + 1)] are not expressed: ≤0.4, lowly expressed: >0.4 and ≤1.4, and expressed: >1.4. Thresholds for gene expression [log_2_(RPKM + 1)] are not expressed: ≤1.5, lowly expressed: >1.5 and ≤5.0, and expressed: >5.0.

### Gene expression analysis

Gene expression in hPGCs and GSCs was quantified by analyzing published RNA-seq data (NCBI SRA: SRP057098) as described previously (*2*). Briefly, trimmed RNA-seq reads were mapped to the reference genome (GRCh38/hg38) using TopHat2 with default parameters, and transcript counts were determined by featureCounts. Replicates were evaluated, counts were normalized, and DE of transcripts was evaluated using the R Bioconductor DEseq2 package. Normalized transcript counts were further normalized by transcript length (per kilobase). Transcript annotations in all bioinformatics analyses were based on Ensembl (Release 96), considering protein coding and lncRNAs only.

Gene expression in *SETDB1-AID* and *SETDB1-WT* naïve hESCs was analyzed as described before (*28*). Adaptor sequences were removed by Trim Galore (v0.4.1) using the default parameters, and reads were mapped to the human reference genome (UCSC GRCh38/hg38) using STAR (2.7.1a) (parameters: “--outFilterMismatchNoverLmax 0.05 --outFilterMultimapNmax 50 --outMultimapperOrder Random”) guided by the GENCODE Human Release 30 comprehensive gene annotation. Raw read counts per gene were extracted by the featureCounts function of the Subread package (1.6.2) using the default parameters. Normalized read counts and DE genes were obtained using DEseq2 (1.26.0) in R (3.6.2)/Bioconductor (3.10.1). For all expression analysis, a log_2_(normalized counts +1) transformation was applied. Only “protein_coding” and “lncRNA” genes were retained in subsequent genome-wide analysis.

Gene expression in ex vivo cultured fhPGCs and fGSCs were processed as described for *SETDB1-AID* and *SETDB1-WT* hESCs, except that the adaptor sequences were removed by Flexbar (3.5.0) as specified by NEB (https://github.com/nebiolabs/nebnext-single-cell-rna-seq) with additional options (“--qtrim TAIL --qtrim-format i1.8 -qt 20”) to remove low-quality reads [deposited at Zenodo (7339593)].

Published scRNA-seq data from Wk4-Wk19 human PGCs (GSE63818) (*30*) were downloaded, trimmed using TrimGalore! (www.bioinformatics.babraham.ac.uk/projects/trim_galore/), and aligned to human transcriptome (GENCODE v30) using kallisto 0.45.0 (*73*). DE at the transcript or gene level was evaluated using a likelihood ratio test with Sleuth v0.30.0 (*74*). Expression plots of transcripts per million were generated using ggplot2 3.2.1.

### Western blot analysis

Western blot analysis on Essential 8 hESCs was performed as described previously (*28*). Briefly, 10^5^ frozen cells were resuspended in 50 mM tris-HCl (pH 8.0), 1% SDS, 10 mM EDTA, and 1× protease inhibitor cocktail (Roche) and lysed by 10 min of incubation on ice. Samples were sonicated for five cycles (30 s on/off) in a Bioruptor Plus (Diagenode) and subsequently incubated for 5 min at 95°C at 1300 rpm in a shaker (Eppendorf). In addition, 5 μl of 20% SDS were added before another five cycles of sonification. Cell lysates were cleared through 10 min of centrifugation at 13,000*g*, protein concentrations (determined using the Bicinchoninic Acid Kit, Sigma-Aldrich) were adjusted, and samples were incubated for 5 min at 95°C following the addition of Laemmli buffer. Proteins were separated on 10% polyacrylamide gel using the Mini-PROTEAN system (Bio-Rad) and transferred to an Immobilon-P transfer membrane (Millipore). After blocking in 5% skimmed milk in tris-buffered saline, 0.1% Tween 20 (0.1% TBST) for 1.5 hours, the membrane was cut according to the molecular weight marker and decorated primary antibodies overnight at 4°C (table S1). Membranes were washed three times with 0.1% TBST for 10 min at RT and decorated with secondary antibodies conjugated to fluorophores or horseradish peroxidase (HRP; table S1) for visualization on the Odyssey CLx (IRDye 680RD, LI-COR) and Western Detection System (GE Healthcare), respectively. The Western blotting reagent (Merck) was used to generate luminescence from the HRP-coupled antibodies.

For Western blots on purified murine and human PGCs and GSCs, 5000 FACS-purified cells were incubated in Laemmli buffer for 5 min on ice and 5 min at 95°C and 1300 rpm in shaker (Eppendorf). Subsequently, samples were sonicated for five cycles (30-s on/off) in a Bioruptor Plus and incubated again for 5 min at 95°C and 1300 rpm in an Eppendorf shaker. The lysates were analyzed on 12.5% polyacrylamide gels as described above.

### Immunofluorescence microscopy

For immunofluorescence microscopy on naïve or Essential 8 hESCs, cells were grown on mouse embryonic fibroblast (MEF)–coated or vitronectin-coated ibiTreat eight-well μ-Slides (Ibidi) for 2 days, respectively. Cells were washed three times with PBS and fixed with 4% PFA (Thermo Fisher Scientific) in PBS at RT for 10 min. After three washes in PBS, 10-min permeabilization in 0.25% Triton X-100 (Sigma-Aldrich) in PBS, samples were blocked [0.1% Triton X-100, 5% normal donkey serum (Stratech), and 1% BSA (Sigma-Aldrich)] for 30 min at RT. Primary antibodies were diluted in blocking buffer and incubated with the samples overnight at 4°C (table S1). After washing in 0.1% Triton X-100 in PBS, samples were incubated with Alexa fluorophore (AF-488, AF-568, and/or AF-647)–conjugated secondary antibodies (Invitrogen, used in 1:500 dilutions in the blocking buffer) for 1 hour at RT in the dark. After three washes, the samples were incubated in PBS with 4′,6-diamidino-2-phenylindole (1 μg/ml) for 10 min at RT and imaged using the Leica SP8 upright or inverted scanning confocal microscope and analyzed using Volocity (6.3).

For staining against 5mC, cells were washed three times for 5 min with PBS after fixation and incubated for 10 min with 3 N HCl, which was neutralized with 100 mM tris-HCl (pH 8) for 10 min. Subsequently, cells were washed three times with PBS and decorated with the primary and secondary antibodies as described above.

### Human ESC culture and purification of naïve hESCs

The *SETDB1-AID-HA-T2A-EGFP*; *ER-TIR1*; *NANOS3-tdTomato* (*SETDB1-AID*), *SETDB1 wild type*; *ER-TIR1*; *NANOS3-tdTomato* (*SETDB1-WT*), and parental *NANOS3-tdTomato* (*75*) hESCs were maintained on vitronectin-coated plates in Essential 8 (E8) medium (Thermo Fisher Scientific) at 37°C and 5% CO_2_. Culture medium was changed daily. Cells were passaged every 3 to 5 days through incubation with 0.5 mM EDTA in PBS for 2 to 3 min at 37°C and subsequent manual dissociation of the ESC colonies. Conversion of Essential 8 hESCs into the naïve state as described previously (*41*). Briefly, irradiated CF1 MEFs (Gibco) were seeded on gelatine-coated dishes in MEF medium [10% FCS, penicillin-streptomycin (100 U/ml), and DMEM]. The Essential 8-cultured hESCs were washed with PBS, incubated with 0.25% Trypsin-EDTA (Gibco) for 3 min at 37°C, and dissociated in MEF medium by pipetting. Subsequently, cells were seeded on MEFs and incubated at 37°C and 5% CO_2_ in Essential 8 medium supplemented with 10 μM Rock inhibitor [Y27632 (Tocris Bioscience)] for 16 hours. On the next day, the medium was changed to 5iALF medium published before (*41*) supplemented with ascorbic acid (100 μg/ml; Sigma-Aldrich) and incubated at 37°C with 5% CO_2_ and 5% O_2_ for 9 to 10 days with daily medium changes. After conversion, cells were washed, dissociated with Accutase (Gibco), seeded to new MEF-coated dishes, and incubated for 4 to 5 days in 5iALF medium at 37°C with 5% CO_2_ and 5% O_2_, which was considered the first naïve passage. Subsequently, cells were passaged ever 3 to 5 days. All cell lines were confirmed as mycoplasma negative.

For depletion of SETDB1 in Essential 8 hESCs, *SETDB1-AID*, *SETDB1-WT*, and parental hESCs were seeded on vitronectin-coated plates. After 16 hours, medium was change into Essential 8 medium supplemented with 20 nM tamoxifen (Sigma-Aldrich) and 25 μM IAA or vehicle (1:40,000, 96% ethanol). Cells were washed and harvested after 16 hours, 48 hours, and 6 days through incubation with 0.5 mM EDTA in PBS for 5 min at 37°C. Single-cell suspension was generated through pipetting, and 100,000 cells were counted using electronic cell counter, pelleted by centrifugation, and stored at −80°C.

To analyze the effect of SETDB1 depletion in the naïve state, *SETDB1-AID* and *SETDB1-WT* hESCs of naïve passage one were seeded in 5iALF medium supplemented with ascorbic acid (100 μg/ml), 20 nM tamoxifen (Sigma-Aldrich), and 25 μM IAA and cultured for 4 days. Subsequently, cells were harvested by incubation with Accutase for 3 min at 37°C and subsequent dissociation by pipetting in MEF medium. Cells were pelleted, resuspended (PBS, 3% FCS, and 5 mM EDTA), filtered through a 35-μm cell strainer, and stained with anti-CD75 eFluor 660 (5 μl; eBioscience) and Anti-Human Alkaline Phosphatase BV421 (5 μl; BD Biosciences) for 15 min at RT while rotating in darkness at 10 rpm in an orbital rotor. Subsequently, alkaline phosphatase and CD75 double-positive and double-negative cell populations were purified using SH800Z Cell Sorter (Sony), directly lysed using extraction buffer (PicoPure RNA Isolation Kit, Applied Biosystems), and stored at −80°C until RNA extraction.

### Quantitative real-time PCR analysis

RNA was extracted using the PicoPure RNA Isolation Kit (Applied Biosystems) including a DNase I on-column digest (Qiagen) and used to generate cDNA through the SuperScript III Kit (Invitrogen). qPCR was performed on a QuantStudio 6 Flex Real-Time PCR Systems (Applied Biosystems) using SYBR Green JumpStart Taq ReadyMix (Sigma-Aldrich) and specific primers (table S2). The ΔΔ*C*t method was used for quantification of gene expression.

### SETDB1-AID hESC generation

CRISPR-Cas9–mediated insertion of an *AID-HA-T2A-EGFP*-tag at the 3′ end of the endogenous *SETDB1* locus was conducted as the targeting of the *PRDM14* locus described previously (*76*). Briefly, a guide RNA (gRNA) were chosen close to the STOP codon of the *SETDB1* locus by the Custom Alt-R CRISPR-Cas9 gRNA design tool of Integrated DNA Technologies (https://eu.idtdna.com/site/order/designtool/index/CRISPR_SEQUENCE), annealed, and ligated into the pX300 vector (*77*) digested with Bds1 (table S2). The donor vector harboring the *AID-HA-T2A-EGFP* cassette flanked by the *SETDB1* locus homology arms for homology-directed repair was generated using In-Fusion cloning (Clontech) according to the manufacturer’s recommendations. For selection, the donor vector contained a puromycin resistance cassette flanked by Rox sites, located 3′ of the 3′ *SETDB1* homology arm.

To generate a double-inducible *TIR1* transgene (pPB-EF1α-osTIR1-ERT2), the osTIR1-ERT2 fusion protein was clone into a piggyBac backbone harboring a puromycin resistance cassette under the control of an *EF1*α promoter. The *osTIR1* and *ERT2* cassettes were obtained from constructs published previously (*76*, *78*).

Transgenic hESCs were generated by cotransfecting male *NANOS3-tdTomato* reporter hESCs passage number 56 and cultured in Essential 8 medium with the pX300 (gRNA) and donor vector using the Lonza 4D-Nucleofector. Single hESCs were seed in Essential 8 medium supplemented with 10 μM Rock inhibitor (Y-27632, Tocris bioscience) for 24 hours. Selection was started 2 days after electroporation by supplementing Essential 8 medium with puromycin (0.5 μg/ml; Sigma-Aldrich) and continued for 14 days. Homozygous insertions in individual hESCs clones were confirmed by genotyping and sequencing of the *SETDB1* locus. Cotransfection of *SETDB1-AID* homozygous, *SETDB1-AID* heterozygotes, and wild-type hESCs with pCAG-Dre–internal ribosomal site–hygromycin, pPBase, and pPB-EF1α-osTIR1-ERT2 using the Lonza 4D-Nucleofector allowed the removal of the puromycin resistance cassette from the *SETDB1* locus through transient expression of the Dre recombinase and random insertion of the *TIR1-ERT2* transgene. Cells were selected for 2 days of hygromycin B (50 μg/ml). The removal of the puromycin resistance cassette from the *SETDB1* locus and the insertion of the *TIR1-ERT2* were verified by genotyping.

### Assignment of TEs to promoters

A specific set of TEs, e.g., H3K27me3- or H2aK119ub-occupied TEs, was assigned to the nearest promoter (distance to TSS of <100 kb) using BETA (1.0.7).

### Statistical analysis

Because of the large sample size of our dataset, we used Wilcoxon and chi-square “goodness of fit” effect size as statistical measure for differences in the data (R rstatix package). For the analysis of smaller samples, we used Student’s *t* and chi-square test (R rstatix package).

## Notes

View/request a protocol for this paper from *Bio-protocol*.
