# Supplementary material for: Epigenetic resetting in the human germ line entails histone modification remodeling
Source: Sci Adv. 2023 Jan 18;9(3):eade1257. doi: 10.1126/sciadv.ade1257 (PMC9848478; doi:10.1126/sciadv.ade1257)
Supplement: Supplementary file 1 — Figs. S1 to S11 Tables S1 and S2 [file sciadv.ade1257_sm.pdf]

Science Advances
AAAS

Supplementary Materials for

Epigenetic resetting in the human germ line entails histone modification remodeling

Wolfram H. Gruhn *et al.*

Corresponding author: Wolfram H. Gruhn, w.gruhn@gurdon.cam.ac.uk;
M. Azim Surani, a.surani@gurdon.cam.ac.uk

Sci. Adv. **9**, eade1257 (2023)
DOI: 10.1126/sciadv.ade1257

This PDF file includes:

Figs. S1 to S11
Tables S1 and S2

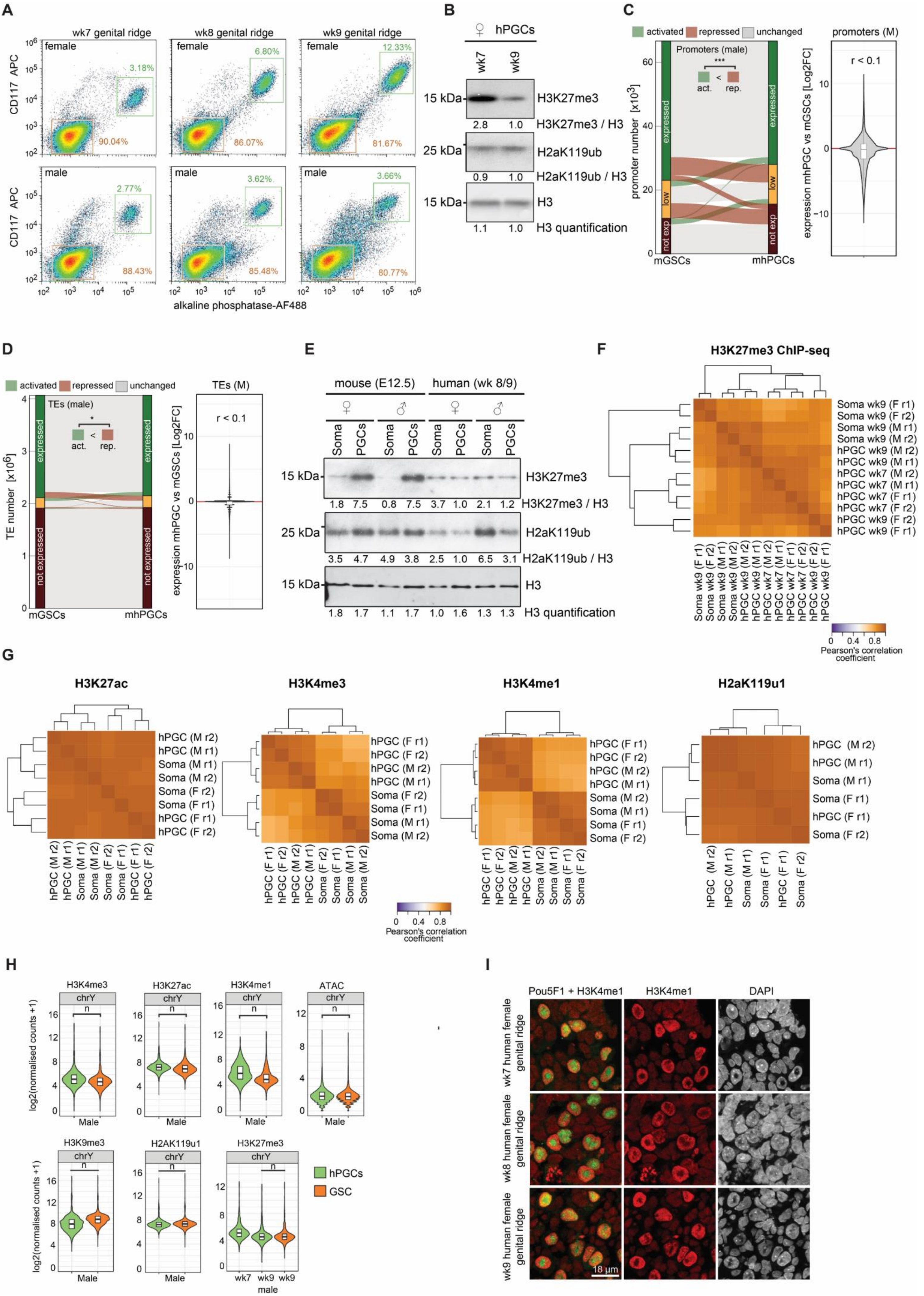
A
wk7 genital ridge
wk8 genital ridge
wk9 genital ridge
female
male
CD117 APC
alkaline phosphatase-AF488
3.18%
90.04%
6.80%
86.07%
12.33%
81.67%
2.77%
88.43%
3.62%
85.48%
3.66%
80.77%
B
♀ hPGCs
wk7
wk9
15 kDa
H3K27me3
2.8
1.0
H3K27me3 / H3
25 kDa
H2aK119ub
0.9
1.0
H2aK119ub / H3
15 kDa
H3
1.1
1.0
H3 quantification
C
activated
repressed
unchanged
Promoters (male)
***
act. < rep.
promoter number [x10^3]
expressed
low
not exp
mGSCs
mhPGCs
promoters (M)
r < 0.1
expression mhPGC vs mGSCs [Log2FC]
D
activated
repressed
unchanged
TEs (male)
*
act. < rep.
TE number [x10^6]
expressed
not expressed
mGSCs
mhPGCs
TEs (M)
r < 0.1
expression mhPGC vs mGSCs [Log2FC]
E
mouse (E12.5)
human (wk 8/9)
♀
♂
Soma
PGCs
15 kDa
H3K27me3
1.8 7.5 0.8 7.5 3.7 1.0 2.1 1.2
H3K27me3 / H3
25 kDa
H2aK119ub
3.5 4.7 4.9 3.8 2.5 1.0 6.5 3.1
H2aK119ub / H3
15 kDa
H3
1.8 1.7 1.1 1.7 1.0 1.6 1.3 1.3
H3 quantification
F
H3K27me3 ChIP-seq
Soma wk9 (F r1)
Soma wk9 (F r2)
Soma wk9 (M r1)
Soma wk9 (M r2)
hPGC wk9 (M r2)
hPGC wk9 (M r1)
hPGC wk7 (M r2)
hPGC wk7 (M r1)
hPGC wk7 (F r1)
hPGC wk7 (F r2)
hPGC wk9 (F r2)
hPGC wk9 (F r1)
Pearson's correlation coefficient
G
H3K27ac
hPGC (M r2)
hPGC (M r1)
Soma (M r1)
Soma (M r2)
Soma (F r2)
Soma (F r1)
hPGC (F r1)
hPGC (F r2)
H3K4me3
hPGC (F r1)
hPGC (F r2)
hPGC (M r2)
hPGC (M r1)
Soma (F r2)
Soma (F r1)
Soma (M r1)
Soma (M r2)
H3K4me1
hPGC (F r1)
hPGC (F r2)
hPGC (M r2)
hPGC (M r1)
Soma (M r2)
Soma (M r1)
Soma (F r1)
Soma (F r2)
H2aK119u1
hPGC (M r2)
hPGC (M r1)
Soma (M r1)
Soma (F r1)
hPGC (F r1)
Soma (F r2)
Pearson's correlation coefficient
H
H3K4me3
H3K27ac
H3K4me1
ATAC
H3K9me3
H2AK119u1
H3K27me3
chrY
n
log2(normalised counts +1)
Male
wk7
wk9
male
hPGCs
GSC
I
Pou5F1 + H3K4me1
H3K4me1
DAPI
wk7 human female genital ridge
wk8 human female genital ridge
wk9 human female genital ridge
18 µm

Figure S1: Epigenetic and transcriptomic state of gonadal hPGCs and adjacent gonadal somatic cells.

A. FACS analysis depicting the proportion of tissue-nonspecific alkaline phosphatase (AP) positive (+) and CD117 positive hPGCs in male and female genital ridges at week 7, 8 and 9 of embryonic development.

B. Western Blot comparing H3K27me3 and H2aK119ub levels in week 7 and week 9 FACS-purified fhPGCs. Quantification of H3 and quantification relative to H3 for H3K27me3 and H2aK119ub are shown.

C-D. Comparison of gene (**C**) and TE (**D**) expression between mhPGCs and mGSCs. Genes / TEs were grouped according to their expression level into i.) expressed (dark green), ii.) lowly expressed (orange), and iii.) not expressed genes (dark red) (see Methods). No broad expression change (grey), reduced expression (red) or elevated expression (green) in hPGCs are indicated (left panel). Wilcoxon effect size is shown. Violine plot depicting expression changes of 66,495 human transcripts (**C**) and 3,040,811 annotated TEs (**D**) in mhPGCs relative to gonadal somatic cells (right panel). r = Wilcoxon effect size

E. Western Blot of PGCs and gonadal somatic cells, FACS-purified from male and female E12.5 GOF mouse embryos, week 8 female and 9 male human genital ridges. Levels of H3K27me3, H2aK119ub and H3 were analysed. Quantification of H3 and quantification relative to H3 for H3K27me3 and H2aK119ub are shown.

F/G. Pearson correlation of peak distribution of individual ChIP-seq experiments targeting the indicated histone modifications

H. Spike-in normalized read counts falling into 5-kb bins covering the Y chromosomes for the indicated ChIP-seq experiments on male hPGCs and gonadal somatic cells. ATAC-seq reads were analysed in 1-kb bins. Wilcoxon effect size is shown.

I. Immunofluorescence staining of POU5F1 (green) and H3K4me1 (red) on wk7-9 female human genital ridges. DAPI staining is shown to indicate the location of the nuclei.

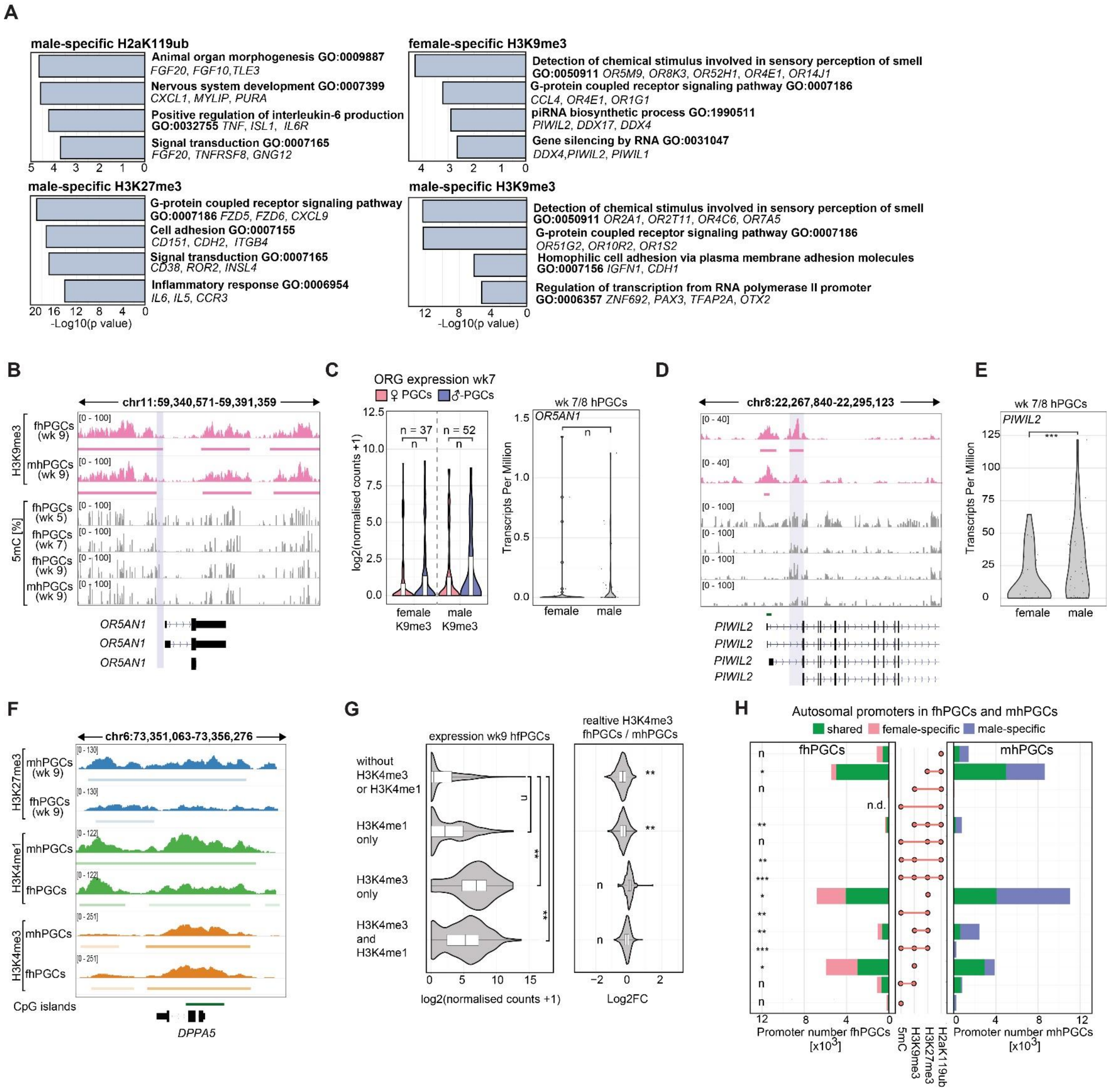

Figure S2: Epigenetic promoter regulation in male and female hPGCs.

A. Gene ontology enrichment analysis of genes associated with promoters showing female-specific occupancy of H3K9me3, or male-specific occupancy of H3K27me3, H3K9me3 or H2aK119ub in hPGCs.

B. Genome browser view of the *OR5AN1* locus showing the indicated epigenetic modifications in fhPGCs and mhPGCs.

C. Expression of ORGs, sex-specifically occupied by H3K9me3, in female (red) and male (blue) hPGCs, respectively. Wilcoxon effect size is shown (left panel). *OR5AN1* expression in mhPGCs and fhPGCs (right panel). Re-analysis of single cell RNA-seq data (*30*). Sleuth's likelihood ratio test is shown. n, $P > 0.05$

D. Genome browser view of the *PIWIL2* locus. Shown epigenetic modifications in fhPGCs and mhPGCs as indicated in Fig. S2B.

E. *PIWIL2* expression in mhPGCs and fhPGCs. Reanalysis of single cell RNA-seq data[30]. Sleuth's likelihood ratio test is shown. ***. $P < 0.005$

F. Genome browser view of the *DPPA5* locus showing the indicated epigenetic modifications in fhPGCs and mhPGCs.

G. Expression of genes associated with male-specific H3K27me3 promoters, co-occupied by the indicated epigenetic marks in wk9 fhPGCs. Wilcoxon effect size is shown (left panel). Differential H3K4me3 level between fhPGCs and mhPGCs at male-specific H3K27me3 promoters, co-occupied by the indicated epigenetic marks. Wilcoxon effect size of differential H3K4me3 levels in fhPGCs and mhPGCs (right panel).

H. Number of promoters occupied by the indicated combinations of repressive marks in mhPGCs and fhPGCs. Colour code indicates promoters occupied by the same combination of repressive marks in germ cells of both sexes (green) or specifically male (blue) or female hPGCs (red). Chi-square “goodness-of-fit” derived effect size.

Effect size levels as in Figure 1.

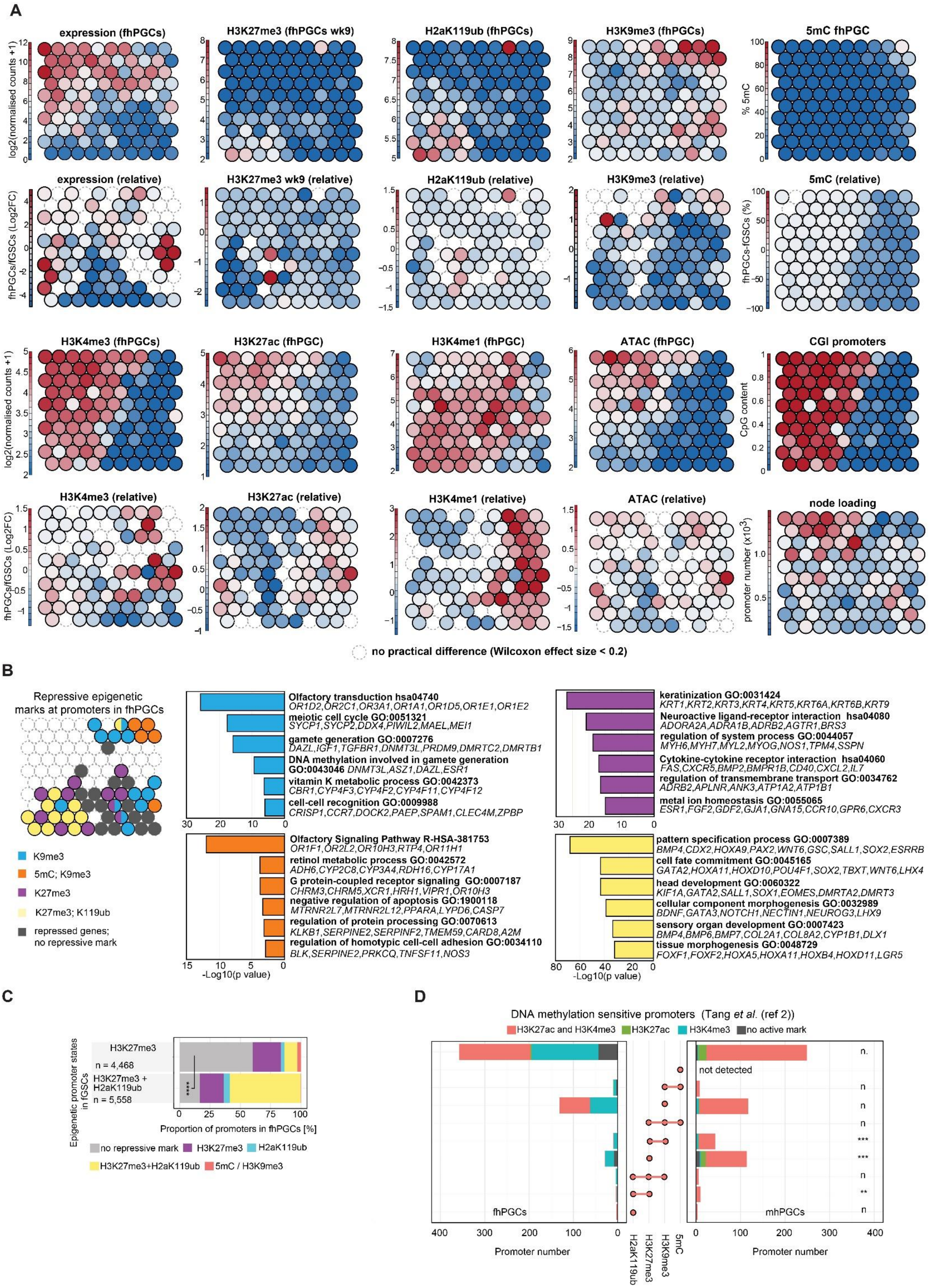
A
expression (fhPGCs)
H3K27me3 (fhPGCs wk9)
H2aK119ub (fhPGCs)
H3K9me3 (fhPGCs)
5mC fhPGC
log2(normalised counts +1)
% 5mC
expression (relative)
H3K27me3 wk9 (relative)
H2aK119ub (relative)
H3K9me3 (relative)
5mC (relative)
fhPGCs/fGSCs (Log2FC)
fhPGCs-fGSCs (%)
H3K4me3 (fhPGCs)
H3K27ac (fhPGC)
H3K4me1 (fhPGC)
ATAC (fhPGC)
CGI promoters
CpG content
H3K4me3 (relative)
H3K27ac (relative)
H3K4me1 (relative)
ATAC (relative)
node loading
promoter number (x10^3)
no practical difference (Wilcoxon effect size < 0.2)
B
Repressive epigenetic marks at promoters in fhPGCs
K9me3
5mC; K9me3
K27me3
K27me3; K119ub
repressed genes; no repressive mark
Olfactory transduction hsa04740
OR1D2,OR2C1,OR3A1,OR1A1,OR1D5,OR1E1,OR1E2
meiotic cell cycle GO:0051321
SYCP1,SYCP2,DDX4,PIWIL2,MAEL,MEI1
gamete generation GO:0007276
DAZL,IGF1,TGFBR1,DNMT3L,PRDM9,DMRTC2,DMRTB1
DNA methylation involved in gamete generation GO:0043046 DNMT3L,ASZ1,DAZL,ESR1
vitamin K metabolic process GO:0042373
CBR1,CYP4F3,CYP4F2,CYP4F11,CYP4F12
cell-cell recognition GO:0009988
CRISP1,CCR7,DOCK2,PAEP,SPAM1,CLEC4M,ZPBP
keratinization GO:0031424
KRT1,KRT2,KRT3,KRT4,KRT5,KRT6A,KRT6B,KRT9
Neuroactive ligand-receptor interaction hsa04080
ADORA2A,ADRA1B,ADRB2,AGTR1,BRS3
regulation of system process GO:0044057
MYH6,MYH7,MYL2,MYOG,NOS1,TPM4,SSPN
Cytokine-cytokine receptor interaction hsa04060
FAS,CXCR5,BMP2,BMPR1B,CD40,CXCL2,IL7
regulation of transmembrane transport GO:0034762
ADRB2,APLNR,ANK3,ATP1A2,ATP1B1
metal ion homeostasis GO:0055065
ESR1,FGF2,GDF2,GJA1,GNA15,CCR10,GPR6,CXCR3
Olfactory Signaling Pathway R-HSA-381753
OR1F1,OR2L2,OR10H3,RTP4,OR11H1
retinol metabolic process GO:0042572
ADH6,CYP2C8,CYP3A4,RDH16,CYP17A1
G protein-coupled receptor signaling GO:0007187
CHRM3,CHRM5,XCR1,HRH1,VIPR1,OR10H3
negative regulation of apoptosis GO:1900118
MTRNR2L7,MTRNR2L12,PPARA,LYPD6,CASP7
regulation of protein processing GO:0070613
KLKB1,SERPINE2,SERPINF2,TMEM59,CARD8,A2M
regulation of homotypic cell-cell adhesion GO:0034110
BLK,SERPINE2,PRKCQ,TNFSF11,NOS3
pattern specification process GO:0007389
BMP4,CDX2,HOXA9,PAX2,WNT6,GSC,SALL1,SOX2,ESRRB
cell fate commitment GO:0045165
GATA2,HOXA11,HOXD10,POU4F1,SOX2,TBXT,WNT6,LHX4
head development GO:0060322
KIF1A,GATA2,SALL1,SOX1,EOMES,DMRTA2,DMRT3
cellular component morphogenesis GO:0032989
BDNF,GATA3,NOTCH1,NECTIN1,NEUROG3,LHX9
sensory organ development GO:0007423
BMP4,BMP6,BMP7,COL2A1,COL8A2,CYP1B1,DLX1
tissue morphogenesis GO:0048729
FOXF1,FOXF2,HOXA5,HOXA11,HOXB4,HOXD11,LGR5
-Log10(p value)
C
Epigenetic promoter states in fGSCs
H3K27me3
n = 4,468
H3K27me3 + H2aK119ub
n = 5,558
****
Proportion of promoters in fhPGCs [%]
no repressive mark
H3K27me3
H2aK119ub
H3K27me3+H2aK119ub
5mC / H3K9me3
D
DNA methylation sensitive promoters (Tang et al. (ref 2))
H3K27ac and H3K4me3
H3K27ac
H3K4me3
no active mark
not detected
fhPGCs
mhPGCs
Promoter number
H2aK119ub
H3K27me3
H3K9me3
5mC
n.
n
***
**

Figure S3: Epigenetic promoter regulation in female hPGCs.

A. SOM analysis depicting clustering of transcript levels, H3K27me3, H2aK119ub, H3K9me3, 5mC, H3K4me3, H3K27ac, H3K4me1 promoter occupancy and ATAC-seq signal, in fhPGCs and changes of these features relative to fGSCs. GpC promoter content and promoter number in each SOM node (right, lower panel). For differential levels between fhPGCs and fGSCs paired Wilcoxon effect size (*r*) was determined and only nodes with $r > 0.2$ are shown.

B. Summary of the SOM analysis of repressive epigenetic mark in fhPGCs (left). Gene ontology enrichment analysis of promoters occupied in fhPGCs by H3K9me3 (blue), H3K9me3 and 5mC (orange), H3K27me3 (violet) or H3K27me3 and H2aK119ub (yellow) (right).

C. Comparison of promoters occupied by H3K27me3 and H3K27me3/H2aK119ub in fGSCs with the repressive epigenetic state of these promoters in fhPGCs. Chi-square "goodness-of-fit" test ****: $P < 0.001$

D. Epigenetic state of promoters associated with DNA methylation sensitive genes identified before (*2*) in fhPGCs (left panel) and mhPGCs (right panel). Colour code indicates the co-occupancy with the active marks H3K4me3 (opal), H3K27ac (green), both marks (orange) or none of them (grey). Chi-square "goodness-of-fit" derived effect size (*r*) no practical difference (n): $r < 0.2$, *: $0.2 \leq r < 0.3$, **: $0.3 \leq r < 0.5$, ***: $r \geq 0.5$

A-D. H3K27me3 occupancy was determined in wk9 fhPGCs/fGSCs.

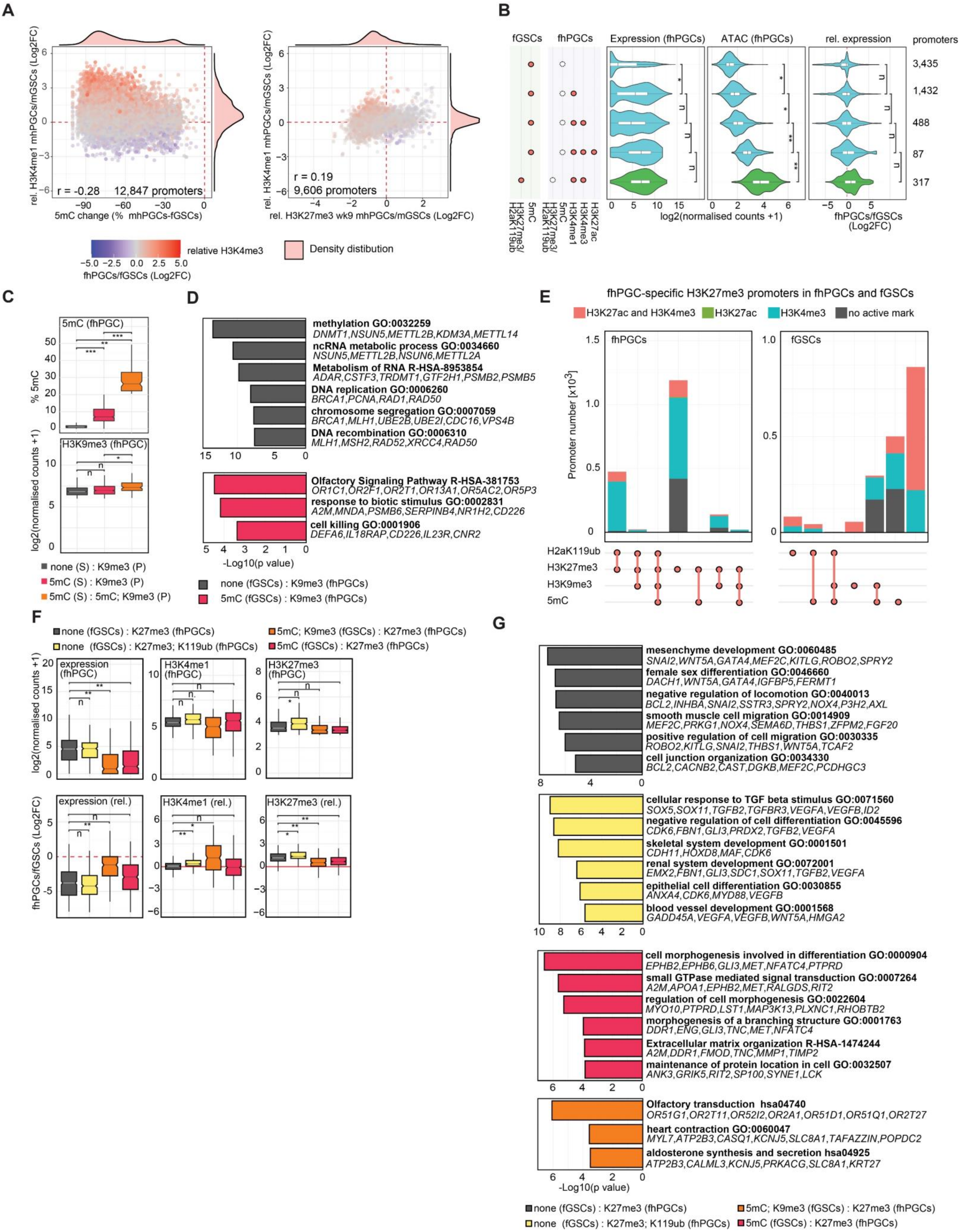
A
rel. H3K4me1 mhPGCs/mGSCs (Log2FC)
r = -0.28 12,847 promoters
5mC change (% mhPGCs-fGSCs)
r = 0.19
9,606 promoters
rel. H3K27me3 wk9 mhPGCs/mGSCs (Log2FC)
relative H3K4me3
fhPGCs/fGSCs (Log2FC)
Density distibution
B
fGSCs
fhPGCs
Expression (fhPGCs)
ATAC (fhPGCs)
rel. expression
promoters
3,435
1,432
488
87
317
H3K27me3/H2aK119ub
5mC
H3K4me1
H3K4me3
H3K27ac
log2(normalised counts +1)
fhPGCs/fGSCs (Log2FC)
C
5mC (fhPGC)
% 5mC
H3K9me3 (fhPGC)
log2(normalised counts +1)
none (S) : K9me3 (P)
5mC (S) : K9me3 (P)
5mC (S) : 5mC; K9me3 (P)
D
methylation GO:0032259
DNMT1,NSUN5,METTL2B,KDM3A,METTL14
ncRNA metabolic process GO:0034660
NSUN5,METTL2B,NSUN6,METTL2A
Metabolism of RNA R-HSA-8953854
ADAR,CSTF3,TRDMT1,GTF2H1,PSMB2,PSMB5
DNA replication GO:0006260
BRCA1,PCNA,RAD1,RAD50
chromosome segregation GO:0007059
BRCA1,MLH1,UBE2B,UBE2I,CDC16,VPS4B
DNA recombination GO:0006310
MLH1,MSH2,RAD52,XRCC4,RAD50
Olfactory Signaling Pathway R-HSA-381753
OR1C1,OR2F1,OR2T1,OR13A1,OR5AC2,OR5P3
response to biotic stimulus GO:0002831
A2M,MNDA,PSMB6,SERPINB4,NR1H2,CD226
cell killing GO:0001906
DEFA6,IL18RAP,CD226,IL23R,CNR2
-Log10(p value)
none (fGSCs) : K9me3 (fhPGCs)
5mC (fGSCs) : K9me3 (fhPGCs)
E
fhPGC-specific H3K27me3 promoters in fhPGCs and fGSCs
H3K27ac and H3K4me3
H3K27ac
H3K4me3
no active mark
fhPGCs
fGSCs
Promoter number [x10³]
H2aK119ub
H3K27me3
H3K9me3
5mC
F
none (fGSCs) : K27me3 (fhPGCs)
none (fGSCs) : K27me3; K119ub (fhPGCs)
5mC; K9me3 (fGSCs) : K27me3 (fhPGCs)
5mC (fGSCs) : K27me3 (fhPGCs)
expression (fhPGC)
H3K4me1 (fhPGC)
H3K27me3 (fhPGC)
log2(normalised counts +1)
expression (rel.)
H3K4me1 (rel.)
H3K27me3 (rel.)
fhPGCs/fGSCs (Log2FC)
G
mesenchyme development GO:0060485
SNAI2,WNT5A,GATA4,MEF2C,KITLG,ROBO2,SPRY2
female sex differentiation GO:0046660
DACH1,WNT5A,GATA4,IGFBP5,FERMT1
negative regulation of locomotion GO:0040013
BCL2,INHBA,SNAI2,SSTR3,SPRY2,NOX4,P3H2,AXL
smooth muscle cell migration GO:0014909
MEF2C,PRKG1,NOX4,SEMA6D,THBS1,ZFPM2,FGF20
positive regulation of cell migration GO:0030335
ROBO2,KITLG,SNAI2,THBS1,WNT5A,TCAF2
cell junction organization GO:0034330
BCL2,CACNB2,CAST,DGKB,MEF2C,PCDHGC3
cellular response to TGF beta stimulus GO:0071560
SOX5,SOX11,TGFB2,TGFBR3,VEGFA,VEGFB,ID2
negative regulation of cell differentiation GO:0045596
CDK6,FBN1,GLI3,PRDX2,TGFB2,VEGFA
skeletal system development GO:0001501
CDH11,HOXD8,MAF,CDK6
renal system development GO:0072001
EMX2,FBN1,GLI3,SDC1,SOX11,TGFB2,VEGFA
epithelial cell differentiation GO:0030855
ANXA4,CDK6,MYD88,VEGFB
blood vessel development GO:0001568
GADD45A,VEGFA,VEGFB,WNT5A,HMGA2
cell morphogenesis involved in differentiation GO:0000904
EPHB2,EPHB6,GLI3,MET,NFATC4,PTPRD
small GTPase mediated signal transduction GO:0007264
A2M,APOA1,EPHB2,MET,RALGDS,RIT2
regulation of cell morphogenesis GO:0022604
MYO10,PTPRD,LST1,MAP3K13,PLXNC1,RHOBTB2
morphogenesis of a branching structure GO:0001763
DDR1,ENG,GLI3,TNC,MET,NFATC4
Extracellular matrix organization R-HSA-1474244
A2M,DDR1,FMOD,TNC,MMP1,TIMP2
maintenance of protein location in cell GO:0032507
ANK3,GRIK5,RIT2,SP100,SYNE1,LCK
Olfactory transduction hsa04740
OR51G1,OR2T11,OR52I2,OR2A1,OR51D1,OR51Q1,OR2T27
heart contraction GO:0060047
MYL7,ATP2B3,CASQ1,KCNJ5,SLC8A1,TAFAZZIN,POPDC2
aldosterone synthesis and secretion hsa04925
ATP2B3,CALML3,KCNJ5,PRKACG,SLC8A1,KRT27
-Log10(p value)
none (fGSCs) : K27me3 (fhPGCs)
none (fGSCs) : K27me3; K119ub (fhPGCs)
5mC; K9me3 (fGSCs) : K27me3 (fhPGCs)
5mC (fGSCs) : K27me3 (fhPGCs)

Figure S4: Putative epigenetic compensation for the loss of DNA methylation in fhPGCs.

A. Scattered plot depicting the correlation between changes of promoter DNA methylation and promoter H3K4me1 levels (left) or changes promoter H3K27me3 and H3K4me1 levels (right). Differential 5mC levels were determined between mhPGCs and fGSCs, differential H3K27me3 and H3K4me1 levels were determined between mhPGCs and mGSCs. Colour code indicates changes in H3K4me3 promoter levels between mhPGCs and mGSCs (Log2 FC mhPGCs/mGSCs). Pearson correlation coefficient (r) is shown. Promoters co-occupied by H3K27me3 and 5mC in mhPGCs or GSCs were excluded from the analysis. Density distribution is shown on each axis.

B. Expression and chromatin accessibility of promoters specifically occupied by 5mC (opal) or H3K27me3/H2aK119ub (green) in fGSCs and marked by the indicated epigenetic modification specifically in fhPGCs. The analysed promoters harboured no detectable repressive marks in fhPGCs and were only occupied by the indicated repressive marks in fGSCs. Wilcoxon effect size is shown.

C. DNA methylation and H3K9me3 levels of fhPGC-specific H3K9me3 promoters occupied in fGSCs (S) by no repressive mark (grey) or 5mC (red). Promoters specifically co-occupied by H3K9me3 and 5mC in fhPGCs (P) are depicted in orange. Wilcoxon effect size is shown.

D. Gene ontology enrichment analysis of promoters with fhPGC-specific H3K9me3 occupancy and no repressive marks (grey) or 5mC occupancy (red) in somatic cells.

E. Epigenetic state of fhPGC-specific H3K27me3 promoters in fhPGCs. Depicted are combinations of repressive epigenetic marks found at these promoters in fhPGCs (left) and fGSCs (right). Colour code indicates the co-occupancy with the active marks H3K4me3 (opal), H3K27ac (green), both marks (orange) or none of them (grey).

F-G. Expression and epigenetic state (**F**) and gene ontology enrichment analysis (**G**) of promoters, not occupied by a repressive mark in somatic cells and marked by H3K27me3 (grey) or H3K27me3 and H2aK119ub (yellow) in fhPGCs. In addition, promoters are shown occupied by 5mC (red) or 5mC and H3K9me3 (orange) in somatic cells and only marked by H3K27me3 in fhPGCs.

A-G. H3K27me3 occupancy was determined in wk9 fhPGCs / fGSCs.

Effect size levels as in Figure 1.

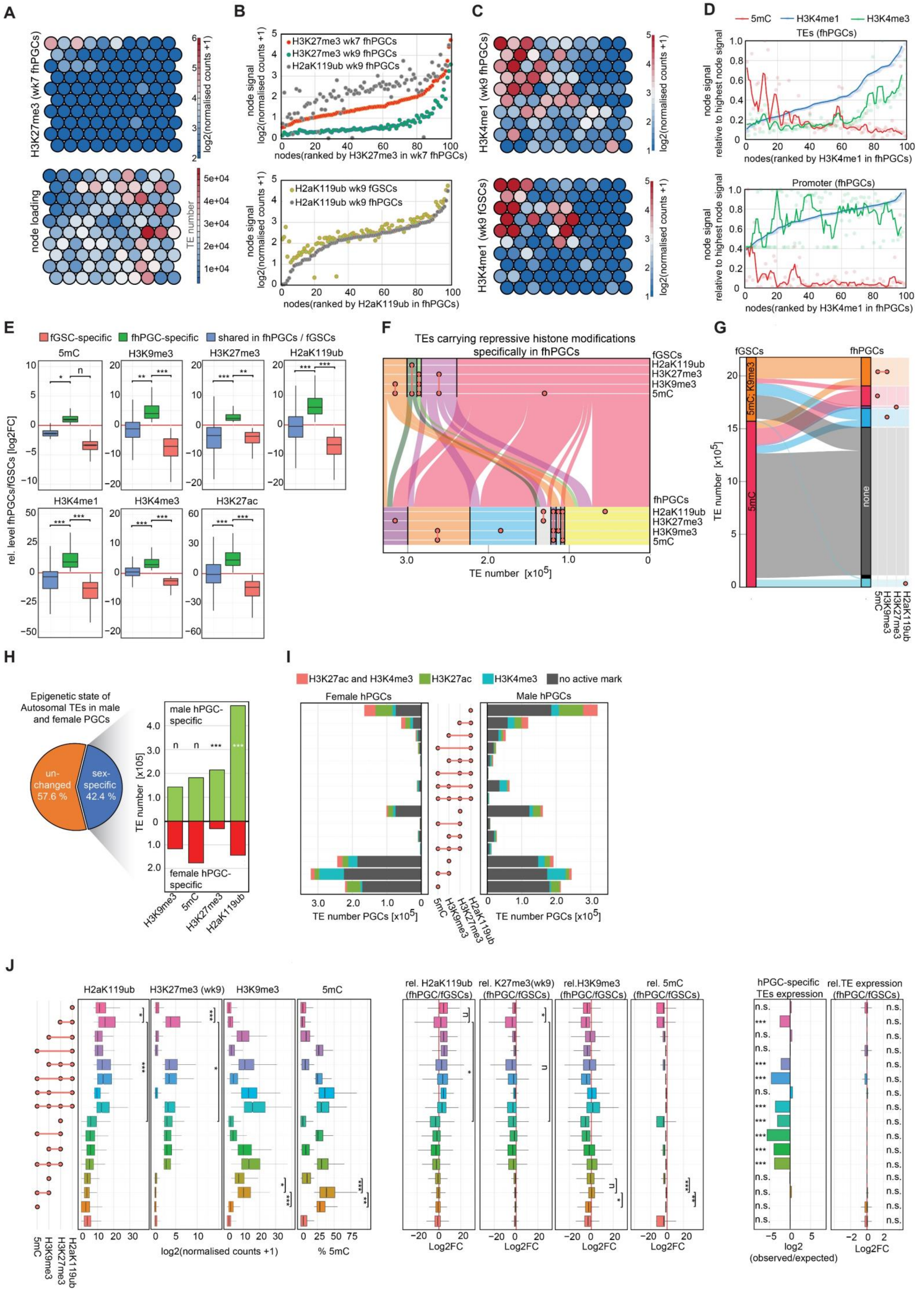
A
B
C
D
H3K27me3 (wk7 fhPGCs)
log2(normalised counts +1)
node loading
TE number
node signal log2(normalised counts +1)
H3K27me3 wk7 fhPGCs
H3K27me3 wk9 fhPGCs
H2aK119ub wk9 fhPGCs
nodes(ranked by H3K27me3 in wk7 fhPGCs)
H2aK119ub wk9 fGSCs
H2aK119ub wk9 fhPGCs
nodes(ranked by H2aK119ub in fhPGCs)
H3K4me1 (wk9 fhPGCs)
H3K4me1 (wk9 fGSCs)
5mC
H3K4me1
H3K4me3
TEs (fhPGCs)
Promoter (fhPGCs)
node signal relative to highest node signal
nodes(ranked by H3K4me1 in fhPGCs)
E
fGSC-specific
fhPGC-specific
shared in fhPGCs / fGSCs
rel. level fhPGCs/fGSCs [log2FC]
H3K9me3
H3K27me3
H2aK119ub
H3K4me1
H3K27ac
F
TEs carrying repressive histone modifications specifically in fhPGCs
fGSCs
fhPGCs
TE number [x10^5]
G
TE number [x10^5]
5mC; K9me3
none
H
Epigenetic state of Autosomal TEs in male and female PGCs
un-changed 57.6 %
sex-specific 42.4 %
male hPGC-specific
female hPGC-specific
TE number [x105]
I
H3K27ac and H3K4me3
no active mark
Female hPGCs
Male hPGCs
TE number PGCs [x10^5]
J
H3K27me3 (wk9)
log2(normalised counts +1)
% 5mC
rel. H2aK119ub (fhPGC/fGSCs)
rel. K27me3(wk9) (fhPGC/fGSCs)
rel.H3K9me3 (fhPGC/fGSCs)
rel. 5mC (fhPGC/fGSCs)
Log2FC
hPGC-specific TEs expression
log2 (observed/expected)
rel.TE expression (fhPGC/fGSCs)

Figure S5: Characterisation of the epigenetic environment at TEs in hPGCs.

A. SOM analysis of TEs depicting H3K27me3 occupancy in wk7 fhPGCs (upper panel) and TE loading in all SOM nodes shown in Fig. 5A, Fig. S5A, C (lower panel).

B. TE SOM node average values for H3K27me3 in wk7 and wk9 fhPGCs and H2aK119ub in wk9 fhPGCs. Nodes were ranked by the H3K27me3 occupancy in wk7 fhPGCs (upper panel). TE SOM node average values for H2aK119ub in fhPGCs and fGSCs. Nodes were ranked by the H2aK119ub occupancy in fhPGCs (lower panel).

C.TE SOM analysis depicting H3K4me1 occupancy in fhPGCs and fGSCs.

D. SOM analysis on TEs (upper panel) and promoters (lower panel) in fhPGCs. SOM node average values for H3K4me1 and H3K4me3 and 5mC are depicted. Nodes were ranked by the H3K4me1 occupancy in fhPGCs.

E. Relative occupancy levels of the indicated modifications in fhPGCs/fGSCs at TEs occupied by the corresponding modification specifically in fhPGCs (green), or fGSCs (red) or in both cell types (blue). Wilcoxon effect size (*r*). no practical difference (n): $r < 0.2$, *: $0.2 \leq r < 0.3$, **: $0.3 \leq r < 0.5$, ***: $r \geq 0.5$

F. Analysis of TEs specifically occupied by H3K9me3, H2aK119ub or H3K27me3 in fhPGCs while exhibiting occupancy with another repressive epigenetic mark in fGSCs. For simplicity only epigenetic state changes that are found in more the 1% of the total analysed TEs (370,102) are shown.

G. Alluvial plot depicting the repressive chromatin modification detected in fhPGCs at TEs marked in somatic cells by 5mC or 5mC/H3K9me3.

H. Pie chart depicting the proportion of autosomal TEs in male and female hPGCs harbouring the same or sex-specific repressive chromatin profiles (left panel). Number of TEs occupied by the indicated marks sex-specifically (right panel). H3K27me3 occupancy was determined in wk9 fhPGCs / mhPGCs. Chi-square “goodness-of-fit” derived effect size. no practical difference (n): $r < 0.2$, *: $0.2 \leq r < 0.3$, **: $0.3 \leq r < 0.5$, ***: $r \geq 0.5$

I. Number of TEs occupied by the indicated combinations of repressive marks in female and male hPGCs. Colour code indicates the co-occupancy with the active marks H3K4me3 (opal), H3K27ac (green), both marks (orange) or none of them (grey). H3K27me3 occupancy was determined in wk9 fhPGCs / mhPGCs.

J. Absolute and relative (fhPGCs/fGSCs) levels of the indicated epigenetic marks in TE subgroups associated with the indicated combinations of epigenetic marks in fhPGCs. Wilcoxon effect size with levels as shown in Figure 1. Level of fhPGC-specifically expressed TEs falling into the indicated TE subgroups is shown as Log2 (observed/expected) with hypergeometric test indicating significance of depletion. *** $P < 0.001$, n.s. $P > 0.05$

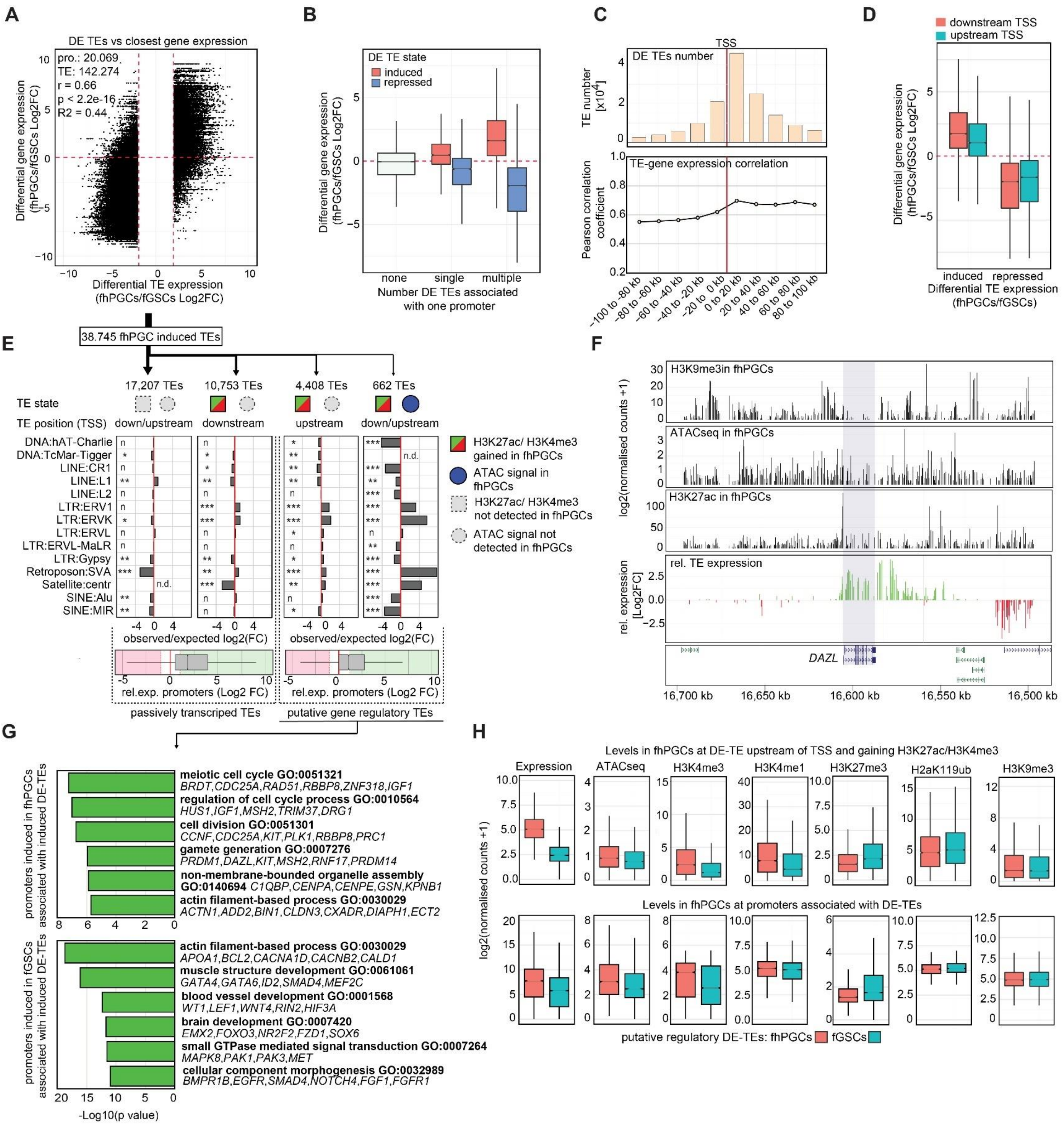

Figure S6: Interconnection between TE and gene expression.

A. Scattered plot analysing the correlation of differential TE expression with differential expression of the closest gene (± 100kb to TSS). Pearson correlation coefficient (r), p value (p)

B. Box plot comparing differential expression (fhPGCs/fGSCs) of genes associated with no, one or multiple differentially expressed TEs (DE-TEs). Genes associated with TEs induced (red) or repressed (blue) in fhPGCs are shown.

C. Number of differentially expressed TEs relative to the TSS of the closest gene (± 100kb TSS) (upper panel). Pearson correlation coefficient of differential gene and DE-TEs expression within the indicated distances to the TSS of the associated gene (lower panel).

D. Box plot comparing differential expression of genes harbouring DE-TEs up- (opal) and downstream (red) of the TSS. DE-TEs are segregated into induced or repressed in fhPGCs relative to fGSCs.

E. Enrichment of TE families within passively transcribed DE-TEs (harbour no H3K27ac or H3K4me3 or showing H3K27ac/ H3K4me3 in fhPGCs downstream of a genic promoter), and putative regulatory DE-TEs (fhPGC-specific H3K27ac/H3K4me3 upstream of a genic promoter or fhPGC-specific H3K27ac/H3K4me3 in the presence of an ATAC-seq signal) induced in fhPGCs (upper panel). Differential expression of genes associated with the indicated subgroups of DE-TEs (lower panel). TE position was determined relative to closest genic TSS (± 100kb). TE enrichment was derived from the observed relative to the expected TE number. Chi-square "goodness-of-fit" derived effect size. no practical difference (n): $r < 0.2$, *: $0.2 \leq r < 0.3$, **: $0.3 \leq r < 0.5$, ***: $r \geq 0.5$

F. Depiction of the indicated histone modifications, ATAC-seq signal, and differential expression of all DE-TEs in the vicinity of the *DAZL* locus (-100kb TSS - +100 kb TTS).

G. Gene ontology enrichment analysis of promoters induced in fhPGC or fGSCs associated with putative regulatory induced DE-TEs.

H. Comparison of the epigenetic environment of putative regulatory DE-TEs (upper panels) induced in fhPGCs (red) or fGSCs (opal) and the genic promoters associated with these DE-TEs (lower panels). H3K27me3 occupancy was determined in wk9 fhPGCs / fGSCs.

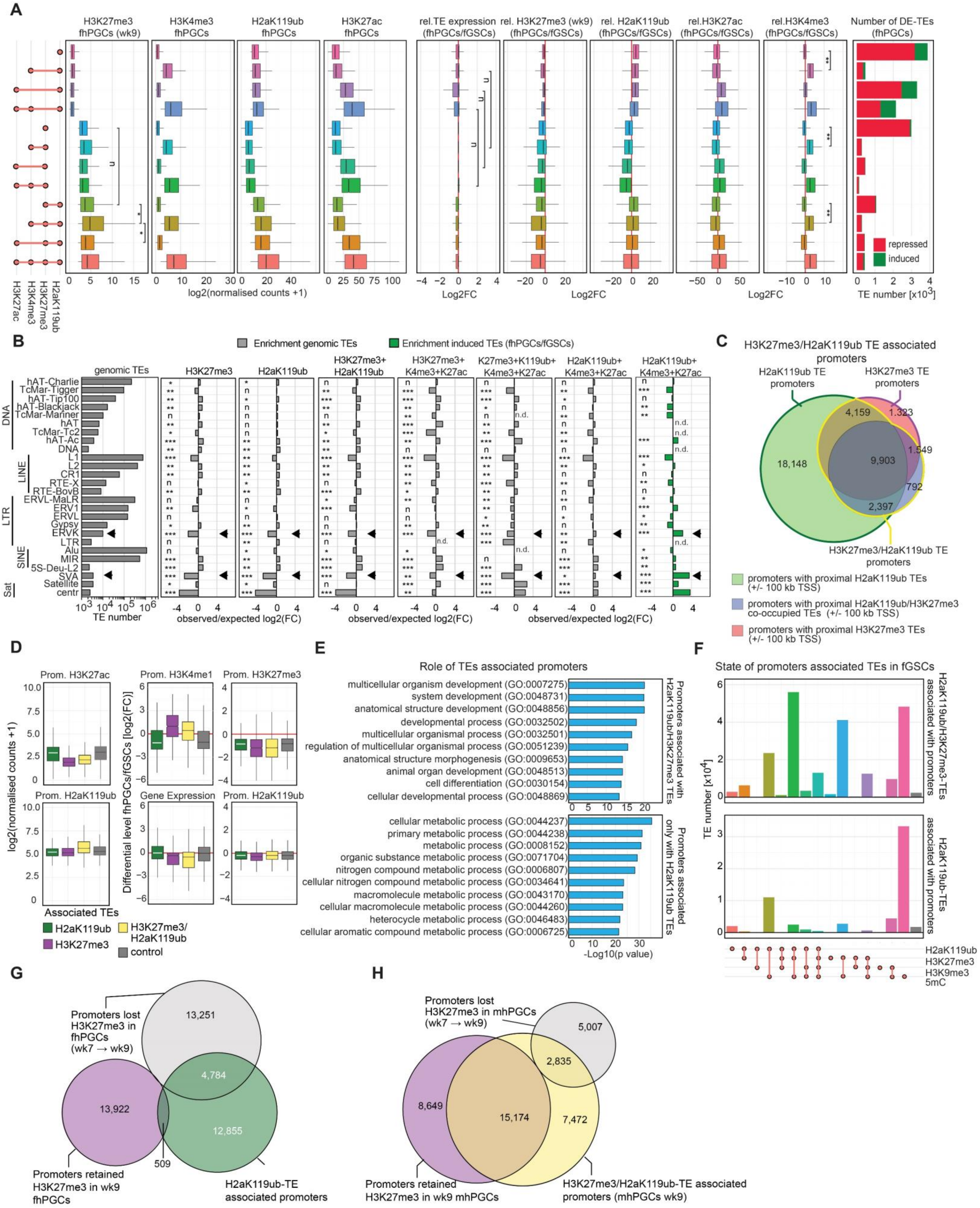

A
H3K27me3 fhPGCs (wk9)
H3K4me3 fhPGCs
H2aK119ub fhPGCs
H3K27ac fhPGCs
log2(normalised counts +1)
rel.TE expression (fhPGCs/fGSCs)
rel. H3K27me3 (wk9) (fhPGCs/fGSCs)
rel. H2aK119ub (fhPGCs/fGSCs)
rel.H3K27ac (fhPGCs/fGSCs)
rel.H3K4me3 (fhPGCs/fGSCs)
Log2FC
Number of DE-TEs (fhPGCs)
repressed
induced
TE number [x10^3]
H3K27ac
H3K4me3
H3K27me3
H2aK119ub
B
Enrichment genomic TEs
Enrichment induced TEs (fhPGCs/fGSCs)
genomic TEs
H3K27me3
H2aK119ub
H3K27me3+ H2aK119ub
H3K27me3+ K4me3+K27ac
K27me3+K119ub+ K4me3+K27ac
H2aK119ub+ K4me3+K27ac
H2aK119ub+ K4me3+K27ac
DNA
LINE
LTR
SINE
Sat
hAT-Charlie
TcMar-Tigger
hAT-Tip100
hAT-Blackjack
TcMar-Mariner
hAT
TcMar-Tc2
hAT-Ac
DNA
L1
L2
CR1
RTE-X
RTE-BovB
ERVL-MaLR
ERV1
ERVL
Gypsy
ERVK
LTR
Alu
MIR
5S-Deu-L2
SVA
Satellite
centr
TE number
observed/expected log2(FC)
n.d.
C
H3K27me3/H2aK119ub TE associated promoters
H2aK119ub TE promoters
H3K27me3 TE promoters
18,148
4,159
1,323
9,903
1,549
792
2,397
H3K27me3/H2aK119ub TE promoters
promoters with proximal H2aK119ub TEs (+/- 100 kb TSS)
promoters with proximal H2aK119ub/H3K27me3 co-occupied TEs (+/- 100 kb TSS)
promoters with proximal H3K27me3 TEs (+/- 100 kb TSS)
D
Prom. H3K27ac
Prom. H3K4me1
Prom. H3K27me3
Prom. H2aK119ub
Gene Expression
Prom. H2aK119ub
log2(normalised counts +1)
Differential level fhPGCs/fGSCs [log2(FC)]
Associated TEs
H2aK119ub
H3K27me3
H3K27me3/ H2aK119ub
control
E
Role of TEs associated promoters
multicellular organism development (GO:0007275)
system development (GO:0048731)
anatomical structure development (GO:0048856)
developmental process (GO:0032502)
multicellular organismal process (GO:0032501)
regulation of multicellular organismal process (GO:0051239)
anatomical structure morphogenesis (GO:0009653)
animal organ development (GO:0048513)
cell differentiation (GO:0030154)
cellular developmental process (GO:0048869)
Promoters associated with H2aK119ub/H3K27me3 TEs
cellular metabolic process (GO:0044237)
primary metabolic process (GO:0044238)
metabolic process (GO:0008152)
organic substance metabolic process (GO:0071704)
nitrogen compound metabolic process (GO:0006807)
cellular nitrogen compound metabolic process (GO:0034641)
macromolecule metabolic process (GO:0043170)
cellular macromolecule metabolic process (GO:0044260)
heterocycle metabolic process (GO:0046483)
cellular aromatic compound metabolic process (GO:0006725)
Promoters associated only with H2aK119ub TEs
-Log10(p value)
F
State of promoters associated TEs in fGSCs
TE number [x10^4]
H2aK119ub/H3K27me3-TEs associated with promoters
H2aK119ub-TEs associated with promoters
H2aK119ub
H3K27me3
H3K9me3
5mC
G
Promoters lost H3K27me3 in fhPGCs (wk7 → wk9)
13,251
4,784
13,922
12,855
509
Promoters retained H3K27me3 in wk9 fhPGCs
H2aK119ub-TE associated promoters
H
Promoters lost H3K27me3 in mhPGCs (wk7 → wk9)
5,007
2,835
8,649
15,174
7,472
Promoters retained H3K27me3 in wk9 mhPGCs
H3K27me3/H2aK119ub-TE associated promoters (mhPGCs wk9)

Figure S7: Characterisation of H3K27me3 and H2aK119ub occupied TEs in fhPGCs.

A. Analysis of the epigenetic and transcriptional state of H3K27me3 and H2aK119ub occupied TEs. Absolute and relative (fhPGCs/fGSCs) levels of expression and occupancy with indicated epigenetic marks in TE subgroups associated with the indicated combinations of epigenetic marks in fhPGCs are depicted. Number differentially expressed TEs between fhPGCs and fGSCs falling in each TE subgroup are shown. Wilcoxon effect size with levels as shown in Figure 1.

B. Enrichment of TE families in TEs marked by H3K27me3, H2aK119ub or both in the presence or absence of H3K27ac and H3K4me3 (grey). Enrichment of TE families in TEs induced in fhPGC and co-occupied by H2aK119ub, H3K27ac and H3K4me3 (green). Arrows indicate evolutionarily young TE families enriched in the TE subgroup occupied by H2aK119ub in the absence of H3K27me3. n.d. = not detected. Chi-square “goodness-of-fit” derived effect size with levels as shown in Figure 1.

C. Overlap of genic promoters associated TEs that are occupied by H2aK119ub (green), H3K27me3 (red) or both (blue).

D. Analysis of genic promoters associated with H3K27me3, H2aK119ub or H3K27me3/ H2aK119ub occupied TEs in the vicinity (TSS +/- 100kb) in fhPGCs. Promoter occupancy with H3K27ac and H2aK119ub in fhPGCs as well as relative levels (fhPGCs/fGSCs) of H3K4me1, H3K27me3, H2aK119ub and expression are depicted.

E. Gene ontology enrichment analysis of promoters associated with TEs marked by H3K27me3 and H2aK119ub or only H2aK119ub in fhPGCs.

F. Repressive epigenetic environment in fGSCs of promoter-proximal TEs identified in fhPGCs. TEs were grouped by their promoter association into i.) only H2aK119ub (lower panel), or ii.) H3K27me3 and H2aK119ub promoter-proximal TEs (upper panel).

G. Overlap between genic promoters that lost (grey) or retained (violet) H3K27me3 between wk7 and 9 in fhPGCs and promoters associated with H2aK119ub occupied TEs (green).

H. Overlap between genic promoters that lost (grey) or retained (violet) H3K27me3 between wk7 and 9 in mhPGCs and promoters associated with H3K27me3/H2aK119ub occupied TEs (yellow).

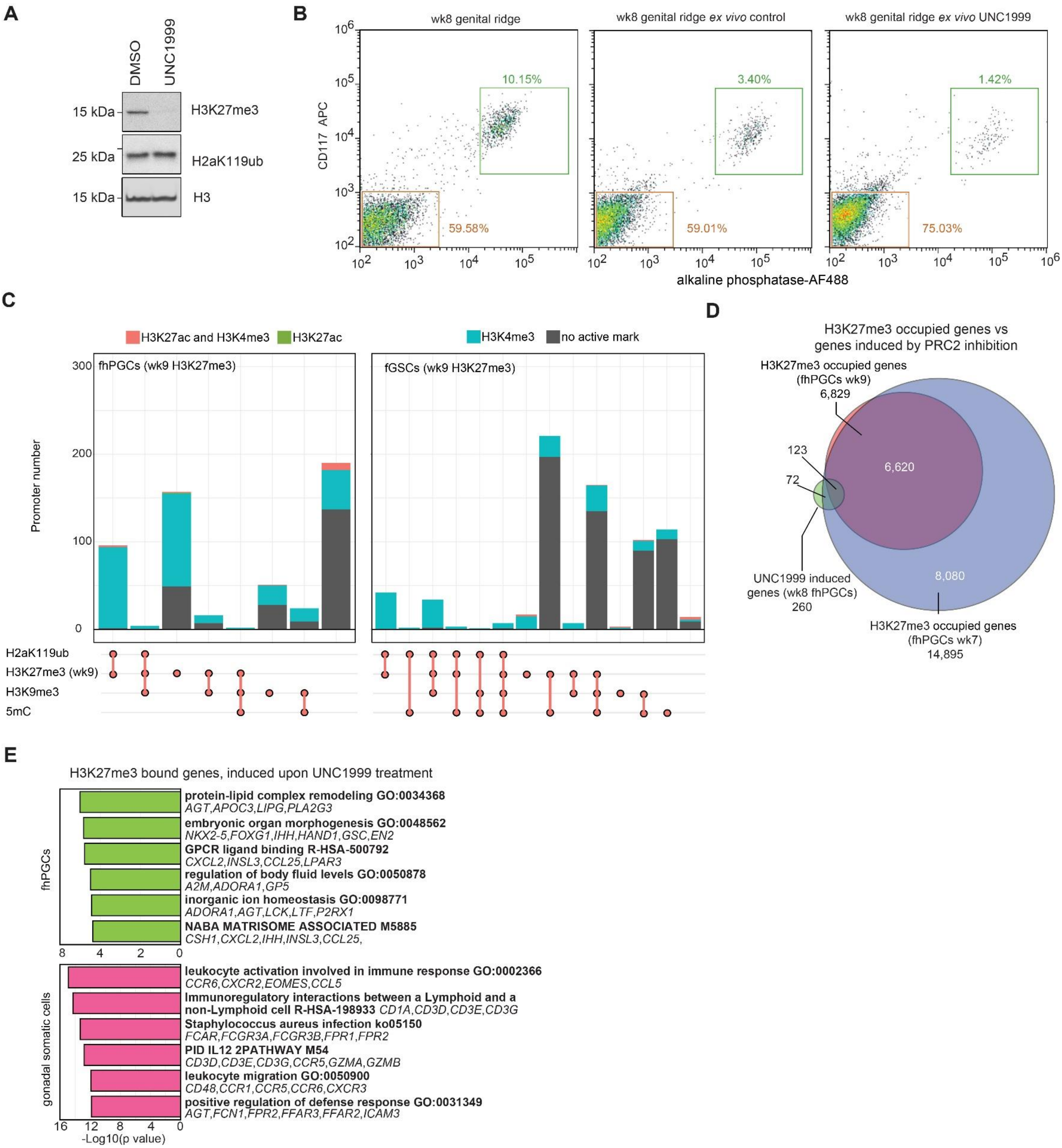
A
DMSO
UNC1999
15 kDa
H3K27me3
25 kDa
H2aK119ub
15 kDa
H3
B
wk8 genital ridge
wk8 genital ridge ex vivo control
wk8 genital ridge ex vivo UNC1999
CD117 APC
10.15%
59.58%
3.40%
59.01%
1.42%
75.03%
alkaline phosphatase-AF488
C
H3K27ac and H3K4me3
H3K27ac
H3K4me3
no active mark
fhPGCs (wk9 H3K27me3)
fGSCs (wk9 H3K27me3)
Promoter number
H2aK119ub
H3K27me3 (wk9)
H3K9me3
5mC
D
H3K27me3 occupied genes vs genes induced by PRC2 inhibition
H3K27me3 occupied genes (fhPGCs wk9) 6,829
123
72
6,620
8,080
UNC1999 induced genes (wk8 fhPGCs) 260
H3K27me3 occupied genes (fhPGCs wk7) 14,895
E
H3K27me3 bound genes, induced upon UNC1999 treatment
fhPGCs
protein-lipid complex remodeling GO:0034368
AGT,APOC3,LIPG,PLA2G3
embryonic organ morphogenesis GO:0048562
NKX2-5,FOXG1,IHH,HAND1,GSC,EN2
GPCR ligand binding R-HSA-500792
CXCL2,INSL3,CCL25,LPAR3
regulation of body fluid levels GO:0050878
A2M,ADORA1,GP5
inorganic ion homeostasis GO:0098771
ADORA1,AGT,LCK,LTF,P2RX1
NABA MATRISOME ASSOCIATED M5885
CSH1,CXCL2,IHH,INSL3,CCL25,
gonadal somatic cells
leukocyte activation involved in immune response GO:0002366
CCR6,CXCR2,EOMES,CCL5
Immunoregulatory interactions between a Lymphoid and a non-Lymphoid cell R-HSA-198933 CD1A,CD3D,CD3E,CD3G
Staphylococcus aureus infection ko05150
FCAR,FCGR3A,FCGR3B,FPR1,FPR2
PID IL12 2PATHWAY M54
CD3D,CD3E,CD3G,CCR5,GZMA,GZMB
leukocyte migration GO:0050900
CD48,CCR1,CCR5,CCR6,CXCR3
positive regulation of defense response GO:0031349
AGT,FCN1,FPR2,FFAR3,FFAR2,ICAM3
-Log10(p value)

Figure S8: H3K27me3 mediates somatic gene repression in fhPGCs.

A. Western Blot on somatic cells purified from human genital ridges *ex vivo* cultured for 10 days in the presence of UNC1999 or control. Membranes were decorated with the indicated antibodies.

B. FACS analysis depicting portions of alkaline phosphatase/CD117 double-positive fhPGCs in non-cultured and *ex vivo* cultured female genital ridges treated with DMSO or 1µM UNC1999 for 10 days. Non-cultured and *ex vivo* cultured genital ridges originate from different and *ex vivo* cultured genital ridges from the same embryo.

C. Repressive marks detected at promoters associated with genes induced in UNC1999 treated fhPGCs. Repressive marks detected in fhPGCs (left panel) or fGSCs (right panel). Colour code indicates the co-occupancy with the active marks H3K4me3 (opal), H3K27ac (green), both marks (orange) or none of both (grey). Note that H3K27me3 promoter occupancy was obtained from wk9 fhPGCs and fGSCs.

D. Overlap between genes induced in UNC1999 treated fhPGCs and genes associated with promoters occupied by H3K27me3 in week 7 or week 9 fhPGCs. Genes were counted to be associated with H3K27me3 when at least one of the annotated promoters was occupied by H3K27me3 in fhPGCs.

E. Gene ontology enrichment analysis of genes associated with H3K27me3 occupied promoters, which were induced in UNC1999 treated fhPGCs (upper panel) or fGSCs (lower panel) relative to control.

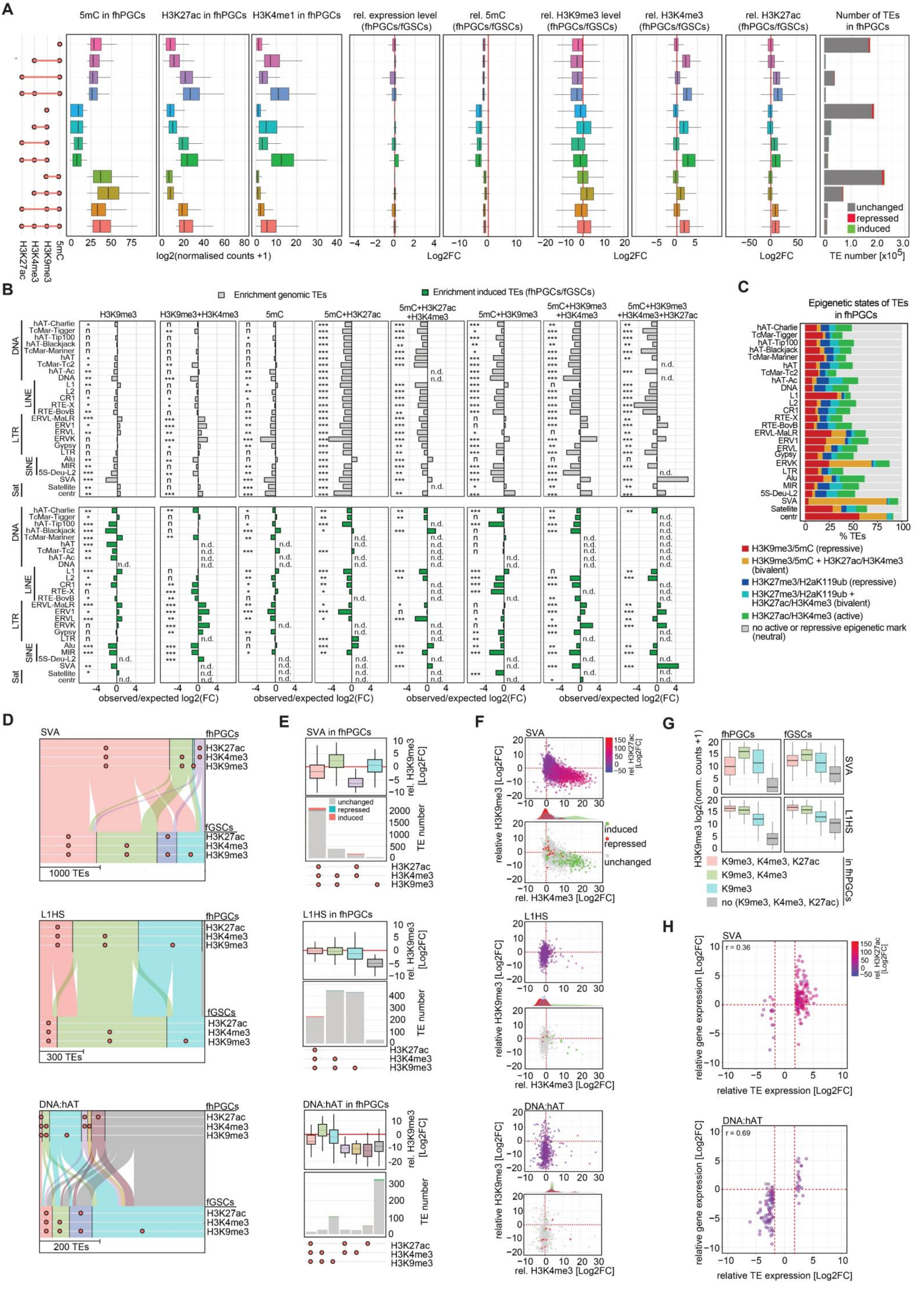

A
5mC in fhPGCs
H3K27ac in fhPGCs
H3K4me1 in fhPGCs
rel. expression level (fhPGCs/fGSCs)
rel. 5mC (fhPGCs/fGSCs)
rel. H3K9me3 level (fhPGCs/fGSCs)
rel. H3K4me3 (fhPGCs/fGSCs)
rel. H3K27ac (fhPGCs/fGSCs)
Number of TEs in fhPGCs
unchanged
repressed
induced
log2(normalised counts +1)
Log2FC
TE number [x10^5]
5mC
H3K9me3
H3K4me3
H3K27ac
B
Enrichment genomic TEs
Enrichment induced TEs (fhPGCs/fGSCs)
H3K9me3
H3K9me3+H3K4me3
5mC
5mC+H3K27ac
5mC+H3K27ac +H3K4me3
5mC+H3K9me3
5mC+H3K9me3 +H3K4me3
5mC+H3K9me3 +H3K4me3+H3K27ac
DNA
LINE
LTR
SINE
Sat
hAT-Charlie
TcMar-Tigger
hAT-Tip100
hAT-Blackjack
TcMar-Mariner
hAT
TcMar-Tc2
hAT-Ac
DNA
L1
L2
CR1
RTE-X
RTE-BovB
ERVL-MaLR
ERV1
ERVL
ERVK
Gypsy
LTR
Alu
MIR
5S-Deu-L2
SVA
Satellite
centr
observed/expected log2(FC)
C
Epigenetic states of TEs in fhPGCs
% TEs
H3K9me3/5mC (repressive)
H3K9me3/5mC + H3K27ac/H3K4me3 (bivalent)
H3K27me3/H2aK119ub (repressive)
H3K27me3/H2aK119ub + H3K27ac/H3K4me3 (bivalent)
H3K27ac/H3K4me3 (active)
no active or repressive epigenetic mark (neutral)
D
SVA
fhPGCs
fGSCs
1000 TEs
L1HS
300 TEs
DNA:hAT
200 TEs
E
SVA in fhPGCs
L1HS in fhPGCs
DNA:hAT in fhPGCs
rel. H3K9me3 [Log2FC]
TE number
F
SVA
L1HS
DNA:hAT
rel. H3K27ac [Log2FC]
relative H3K9me3 [Log2FC]
rel. H3K4me3 [Log2FC]
G
fhPGCs
fGSCs
H3K9me3 log2(norm. counts +1)
K9me3, K4me3, K27ac
K9me3, K4me3
K9me3
no (K9me3, K4me3, K27ac)
in fhPGCs
H
r = 0.36
r = 0.69
relative gene expression [Log2FC]
relative TE expression [Log2FC]

Figure S9: Inefficient euchromatization and retention of H3K9me3 at most evolutionarily young TEs in fhPGCs.

A. Analysis of the epigenetic and transcriptional state of H3K9me3 and 5mC occupied TEs. Absolute and relative (fhPGCs/fGSCs) level of expression and occupancy with indicated epigenetic marks in TE subgroups associated with the indicated combinations of epigenetic marks in fhPGCs are depicted. Number differentially expressed TEs between fhPGCs and fGSCs within the indicated TE subgroups is shown.

B. Enrichment of TE families in TE subgroups marked by H3K9me3, 5mC, or H3K9me3 and 5mC alone or in combination with H3K4me3 or H3K27ac and H3K4me3 in fhPGCs. Enrichment is depicted for TEs annotated within the genome (grey), and for TEs specifically induced in fhPGCs (green). n.d. = not detected. Chi-square "goodness-of-fit" derived effect size. no practical difference (n): $r < 0.2$, *: $0.2 \leq r < 0.3$, **: $0.3 \leq r < 0.5$, ***: $r \geq 0.5$

C. Portion of TEs within major TE families that residing in repressed (only repressive modifications), bivalent (repressive and active modifications) or neutral (no modifications) epigenetic states in fhPGCs. Repressive and bivalent states were subdivided into regulation through A.) H3K9me3 and/or 5mC, and B.) H2aK119ub and/or H3K27me3

D. Alluvial plots comparing the epigenetic states of SVA, L1HS and DNA:hAT elements in fhPGCs and fGSCs. All TEs were selected for occupancy by H3K9me3 in fGSCs.

E. Relative levels of H3K9me3 (fhPGCs/fGSCs) and TE number of SVA, L1HS and DNA:hAT elements grouped according to their epigenetic state in fhPGCs. All TEs were selected for occupancy by H3K9me3 in fGSCs.

F. Comparison of relative levels of H3K9me3 and H3K4me3 (fhPGCs/fGSCs) of SVA, L1HS and DNA:hAT elements. Colour code indicates changes in H3K27ac levels (upper panels) or expression in fhPGCs relative to fGSCs (lower panels). All TEs were selected for being occupied by H3K9me3 in fGSCs. Note, transcriptional induction in fhPGCs is most frequently observed at elements harbouring high levels of H3K4me3 and H3K27ac.

G. Absolute levels of H3K9me3 at SVA and L1HS elements grouped according to their epigenetic state in fhPGCs. All TEs were selected for being occupied by H3K9me3 in fGSCs. Note, SVA but not L1HS elements exhibit reduced H3K9me3 levels when co-occupied by H3K4me3 and H3K27ac.

H. Correlation between relative expression (fhPGCs/fGSCs) of SVA (upper panel) or DNA:hAT elements (lower panel) with expression of the nearest promoter (±100kb TSS). Colour code indicates changes in H3K27ac level between fhPGCs and fGSCs. All shown elements were occupied by H3K9me3 in fGSCs. r = Pearson correlation coefficient

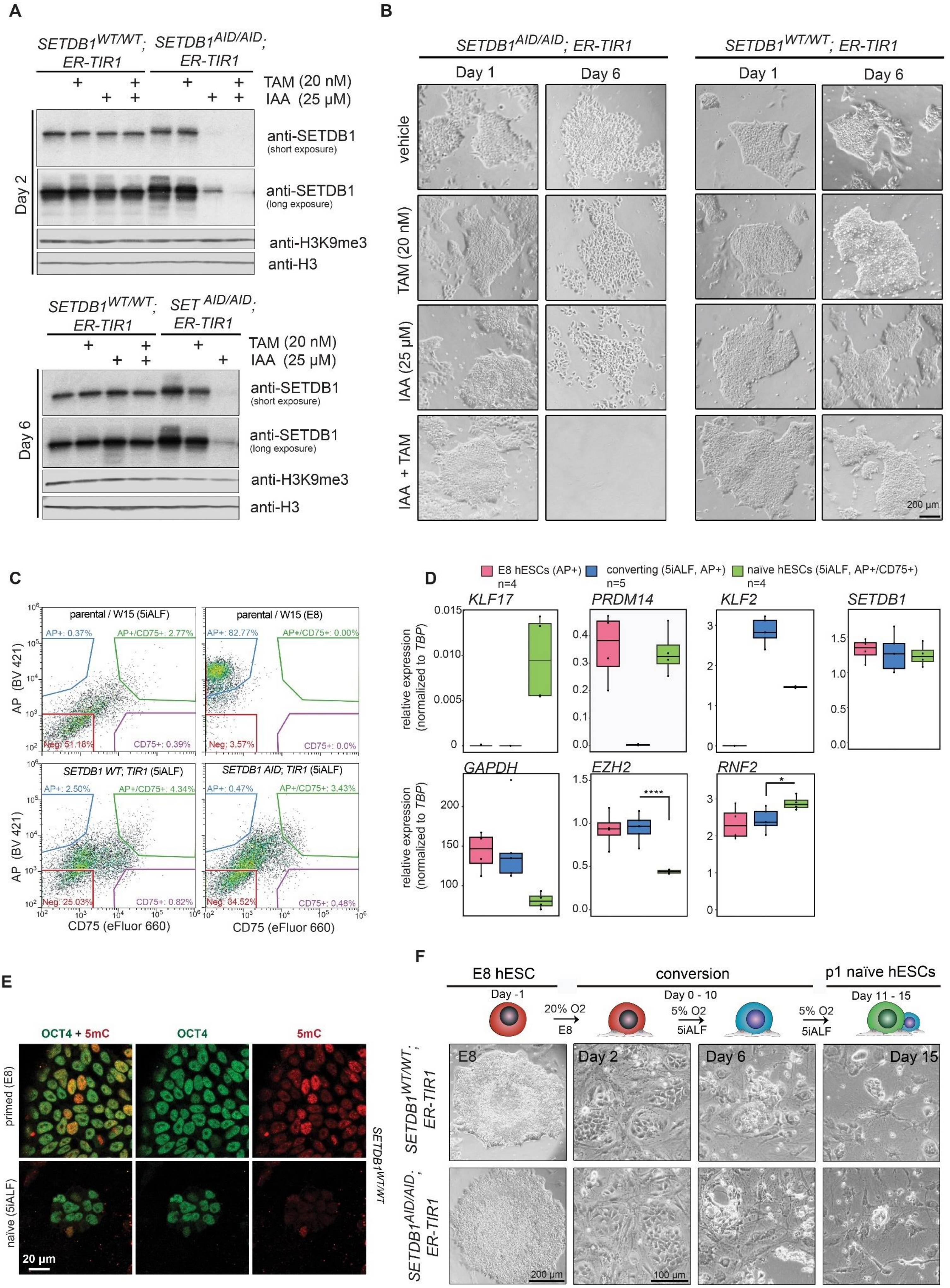
A
SETDB1WT/WT; ER-TIR1
SETDB1AID/AID; ER-TIR1
TAM (20 nM)
IAA (25 µM)
Day 2
anti-SETDB1 (short exposure)
anti-SETDB1 (long exposure)
anti-H3K9me3
anti-H3
SETDB1WT/WT; ER-TIR1
SET AID/AID; ER-TIR1
TAM (20 nM)
IAA (25 µM)
Day 6
anti-SETDB1 (short exposure)
anti-SETDB1 (long exposure)
anti-H3K9me3
anti-H3
B
SETDB1AID/AID; ER-TIR1
SETDB1WT/WT; ER-TIR1
Day 1
Day 6
Day 1
Day 6
vehicle
TAM (20 nM)
IAA (25 µM)
IAA + TAM
200 µm
C
parental / W15 (5iALF)
parental / W15 (E8)
SETDB1 WT; TIR1 (5iALF)
SETDB1 AID; TIR1 (5iALF)
AP+: 0.37%
AP+/CD75+: 2.77%
Neg: 51.18%
CD75+: 0.39%
AP+: 82.77%
AP+/CD75+: 0.00%
Neg: 3.57%
CD75+: 0.0%
AP+: 2.50%
AP+/CD75+: 4.34%
Neg: 25.03%
CD75+: 0.82%
AP+: 0.47%
AP+/CD75+: 3.43%
Neg: 34.52%
CD75+: 0.48%
AP (BV 421)
CD75 (eFluor 660)
D
E8 hESCs (AP+) n=4
converting (5iALF, AP+) n=5
naïve hESCs (5iALF, AP+/CD75+) n=4
KLF17
PRDM14
KLF2
SETDB1
GAPDH
EZH2
RNF2
relative expression (normalized to TBP)
****
*
E
OCT4 + 5mC
OCT4
5mC
primed (E8)
naïve (5iALF)
SETDB1WT/WT
20 µm
F
E8 hESC
conversion
p1 naïve hESCs
Day -1
Day 0 - 10
Day 11 - 15
20% O2
E8
5% O2
5iALF
5% O2
5iALF
SETDB1WT/WT; ER-TIR1
SETDB1AID/AID; ER-TIR1
E8
Day 2
Day 6
Day 15
200 µm
100 µm

Figure S10: Depletion of SETDB1 in hESCs using an inducible degron.

A-B. Western Blot analysis (**A**) and bright field images (**B**) of *SETDB1 wild-type* and *SETDB1-AID* hESCs expressing ER-TIR1 cultured in Essential 8 conditions. Cells were treated for 1 and 6 days with 20 nM Tamoxifen, 25 µM IAA, 20 nM Tamoxifen and 25 µM IAA or vehicle control.

C. FACS analysis on *SETDB1 wild-type* and *SETDB1-AID* naïve hESCs as well as *SETDB1 wild-type* hESCs cultured in Essential 8 conditions stained with anti-alkaline phosphatase (AP)-BV421 and anti- CD75-eFluro660.

D. qRT-PCR analysing the indicated transcripts in FACS purified *SETDB1 wild-type* AP+, CD75+ double-positive naïve hESCs, AP+ converting hESCs, and AP+ hESCs cultured in Essential 8 conditions (n=4, independent technical replicates). Student's t test $p<0.05$ (*), $p<0.001$ (****)

E. Immunofluorescence staining detecting 5mC and OCT4 in passage 2 naïve and Essential 8 *SETDB1 wild-type* hESCs.

F. Bright field images of the conversion of *SETDB1 wild-type* and *SETDB1-AID* cells from Ess8 cultured hESCs to naïve hESCs.

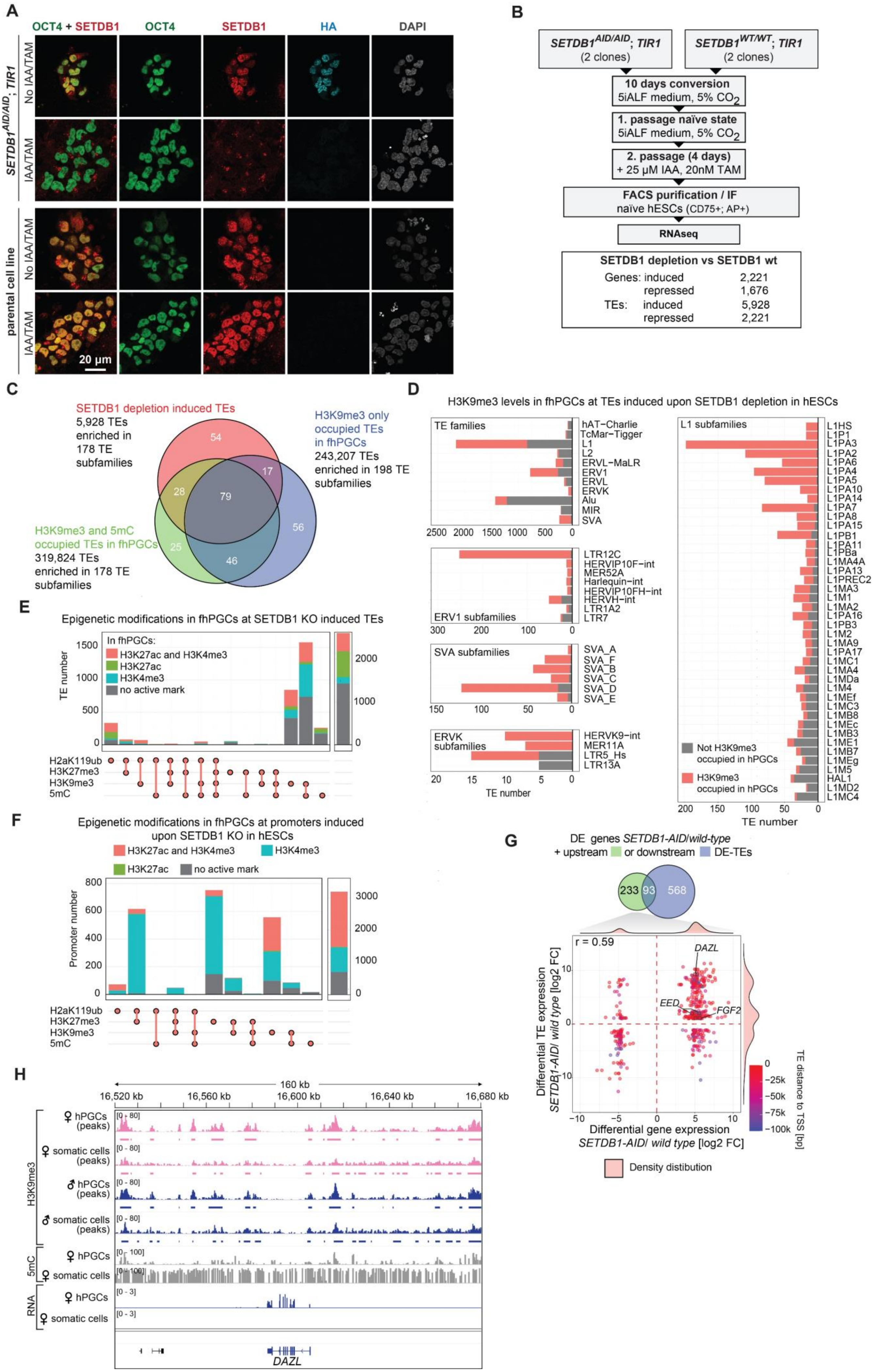

A
OCT4 + SETDB1
OCT4
SETDB1
HA
DAPI
SETDB1^AID/AID; TIR1
No IAA/TAM
IAA/TAM
parental cell line
No IAA/TAM
IAA/TAM
20 µm
B
SETDB1^AID/AID; TIR1 (2 clones)
SETDB1^WT/WT; TIR1 (2 clones)
10 days conversion
5iALF medium, 5% CO2
1. passage naïve state
5iALF medium, 5% CO2
2. passage (4 days)
+ 25 µM IAA, 20nM TAM
FACS purification / IF
naïve hESCs (CD75+; AP+)
RNAseq
SETDB1 depletion vs SETDB1 wt
Genes: induced 2,221
repressed 1,676
TEs: induced 5,928
repressed 2,221
C
SETDB1 depletion induced TEs
5,928 TEs enriched in 178 TE subfamilies
H3K9me3 only occupied TEs in fhPGCs
243,207 TEs enriched in 198 TE subfamilies
H3K9me3 and 5mC occupied TEs in fhPGCs
319,824 TEs enriched in 178 TE subfamilies
54
17
28
79
56
25
46
D
H3K9me3 levels in fhPGCs at TEs induced upon SETDB1 depletion in hESCs
TE families
hAT-Charlie
TcMar-Tigger
L1
L2
ERVL-MaLR
ERV1
ERVL
ERVK
Alu
MIR
SVA
2500 2000 1500 1000 500 0
LTR12C
HERVIP10F-int
MER52A
Harlequin-int
HERVIP10FH-int
HERVH-int
LTR1A2
LTR7
ERV1 subfamilies
300 200 100 0
SVA subfamilies
SVA_A
SVA_F
SVA_B
SVA_C
SVA_D
SVA_E
150 100 50 0
ERVK subfamilies
HERVK9-int
MER11A
LTR5_Hs
LTR13A
20 15 10 5 0
TE number
L1 subfamilies
L1HS
L1P1
L1PA3
L1PA2
L1PA6
L1PA4
L1PA5
L1PA10
L1PA14
L1PA7
L1PA8
L1PA15
L1PB1
L1PA11
L1PBa
L1MA4A
L1PA13
L1PREC2
L1MA3
L1M1
L1MA2
L1PA16
L1PB3
L1M2
L1MA9
L1PA17
L1MC1
L1MA4
L1MDa
L1M4
L1MEf
L1MC3
L1MB8
L1MEc
L1MB3
L1ME1
L1MB7
L1MEg
L1M5
HAL1
L1MD2
L1MC4
Not H3K9me3 occupied in hPGCs
H3K9me3 occupied in hPGCs
200 150 100 50 0
TE number
E
Epigenetic modifications in fhPGCs at SETDB1 KO induced TEs
In fhPGCs:
H3K27ac and H3K4me3
H3K27ac
H3K4me3
no active mark
TE number
1500
1000
500
0
2000
1000
0
H2aK119ub
H3K27me3
H3K9me3
5mC
F
Epigenetic modifications in fhPGCs at promoters induced upon SETDB1 KO in hESCs
H3K27ac and H3K4me3
H3K4me3
H3K27ac
no active mark
Promoter number
800
600
400
200
0
3000
2000
1000
0
H2aK119ub
H3K27me3
H3K9me3
5mC
G
DE genes SETDB1-AID/wild-type
+ upstream or downstream DE-TEs
233
93
568
r = 0.59
DAZL
EED
FGF2
Differential TE expression SETDB1-AID/ wild type [log2 FC]
Differential gene expression SETDB1-AID/ wild type [log2 FC]
-10 -5 0 5 10
TE distance to TSS [bp]
0
-25k
-50k
-75k
-100k
Density distibution
H
160 kb
16,520 kb
16,560 kb
16,600 kb
16,640 kb
16,680 kb
H3K9me3
♀ hPGCs (peaks)
♀ somatic cells (peaks)
♂ hPGCs (peaks)
♂ somatic cells (peaks)
[0 - 80]
5mC
♀ hPGCs
♀ somatic cells
[0 - 100]
RNA
♀ hPGCs
♀ somatic cells
[0 - 3]
DAZL

Figure S11: SETDB1-deposited H3K9me3 safeguards the hypomethylated human genome against evolutionarily young TE expression.

A. Immunofluorescence staining of *SETDB1 wild-type* and *SETDB1-AID* naïve hESCs expressing ER-TIR1. Cells were treated for 4 days with 20 nM Tamoxifen and 25 µM IAA and stained with antibodies detecting HA, OCT4 and SETDB1.

B. Experimental outline of the transcriptomic analysis of SETDB1 depleted naïve hESCs (upper panel) and number of differentially expressed genes and TEs (lower panel). In brief, 2 *SETDB1-AID* and *SETDB1-WT* hESC clones harbouring an *ER-TIR1* transgene were converted into the naïve state and treated with IAA and TAM for 4 days before naïve hESCs were purified by FACS and analysed by RNA-seq. SETDB1 depletion was confirmed by immunofluorescence.

C. Comparison of TE subfamilies enriched for TEs marked in fhPGCs by H3K9me3 alone (blue) or in combination with 5mC (green), with TE subfamilies enriched for transcriptionally induced TEs upon SETDB1 deletion in naïve hESCs (red).

D. TE families and subfamilies of TEs gaining expression upon SETDB1 depletion in naïve hESCs. The colour code indicates whether the corresponding TE locus in fhPGCs is marked by H3K9me3 (red) or not (grey).

E-F. Occupancy of the indicated repressive epigenetic marks in fhPGCs at TEs (**E**) or genes (**F**) transcriptionally induced upon SETDB1 depletion in naïve hESCs. Colour code indicates the co-occupancy with the active marks H3K4me3 (opal), H3K27ac (green), both marks (orange) or none of them (grey). H3K27me3 occupancy was determined in wk9 fhPGCs.

G. Portion of the 3,897 differentially expressed genes between *SETDB1-AID* and wild-type naïve hESCs harbouring differentially expressed TEs upstream (green) or downstream (blue) of the TSS (±100 kb) (upper part). Correlation of expression changes of genes and upstream DE-TEs. Colour code indicates the distance from the nearest TSS (lower part). r = Pearson correlation coefficient.

H. Genome browse snapshots of the *DAZL* locus depicting H3K9me3, 5mC and expression levels in the indicated cell types.

Antibodies used for ChIP-seq						
**Antibody**	**host**	**clonality**	**provider**	**cat. Number**	**lot**	**amount per IP**
anti-H3K4me1	Rabbit	polyclonal	Active Motif	39298	19417002	0.25µl
anti-H3K4me3	Rabbit	polyclonal	Abcam	ab8580	GR273043-4	0.25µg
anti-H3K9me3	Rabbit	polyclonal	Abcam	ab8898	GR257230-1	0.25µg
anti-H3K27ac	Rabbit	polyclonal	Active Motif	39134	20017009	0.25µg
anti-H3K27me3	Rabbit	monoclonal	cell signaling	9733	8	0.5µl
anti-H2AK119ub	Rabbit	polyclonal	cell signaling	8240	6	0.25µl

Antibodies used for Immunofluorescence						
**Antibody**	**host**	**clonality**	**provider**	**cat. number**	**lot number**	**dilution**
anti-GFP	Chicken	Polyclonal	Abcam	ab13970	GR3361051-1	1:1000
anti-OCT4	Mouse	monoclonal	BD Biosciences	611203	8087969	1:500
anti-HA	Rabbit	monoclonal	Cell Signaling Technology	3724		1:500
anti-5mC	Rabbit	polyclonal	Cell Signaling Technology	28692		1:150
anti-SETDB1	Mouse	monoclonal	Abcam	ab107225	GR315074-2	1:500

Antibodies used for FACS						
**Antibody**	**host**	**clonality**	**provider**	**cat. number**	**lot number**	**Volume per staining**
Alexa Fluor 488-conjugated anti-Alkaline Phosphatase	Mouse	Monoclonal	BD Pharmingen	561495	7132712	5ul/sample
APC-conjugated anti-c-KIT	Mouse	Monoclonal	Invitrogen	CD11705	20289675A	5ul/sample
eFluor 660-conjugated anti-CD75	Mouse	Monoclonal	eBioscience	50-0759-42		5ul/sample
BV421-conjugated anti-Human Alkaline Phosphatase	Mouse	Monoclonal	BD biosciences	565624		5ul/sample

Antibodies used for Western Blot						
**Antibody**	**host**	**clonality**	**provider**	**cat. number**	**lot number**	**Volume per staining**
anti-H3	Rabbit	polyclonal	Abcam	ab1791	7132712	1:10000
anti-HA	Rabbit	monoclonal	Cell Signaling Technology	3724	20289675A	1:1000
anti-H3K27me3	Rabbit	monoclonal	Cell Signaling Technology	9733		1:2500
anti-H3K9me3	Rabbit	polyclonal	Abcam	ab8898	GR257230-1	1:5000
anti-H2AK119ub	Rabbit	polyclonal	Cell Signaling Technology	8240		1:2500
anti-SETDB1	Mouse	monoclonal	Abcam	ab107225	GR315074-2	1:1000

Table S1.

Antibodies used in this study

qRT-PCR primers		
Target	Forward (5'→3')	Reverse (5'→3')
EZH2	CCAGACTGGTGAAGAGTTGTTTT	CAAGGGATTTCCATTTCTCG
RNF2	CGACAGCGCACAGACAAG	TTCTCCTTTGCTTCGAAGTTCT
SETDB1	AGGCACGTGGTGGAAGTC	CCACTCACATCTTTTGTCATCC
KLF2	ACCTACACCAAGAGTTCGCATC	CCGTGTGCTTTCGGTAGTGG
KLF17	GCTGCCCAGGATAACGAGAAC	ATCTCTGCGCTGTGAGGAAAG
DNMT3L	GAAGACCTGGACGTCGCATC	AGTGCCTGCTCCTTATGGCT
OTX2	GGGAGAGGACGACGTTCA	TCTGGGTACCGGGTCTTG
DNMT1	ACTGGCGCGATCTGCCCAAC	AGGCTTTGCCGGCTTCCACG
TFCP2L1	AGCTCAAAGTTGTCCTACTGCC	TTCTAACCCAAGCACAGATCCC
PRDM14	CTACCGAGCCCGAGTGGCCTAC	TAGAGCCATCCCGGGACCGCA
GAPDH	CGCTTCGCTCTCTGCTCCTCCTGT	GGTGACCAGGCGCCCAATACGA

CRISPR sgRNA			
gRNA name	Target	Target coordinate (hg38)	gRNA sequence
SETDB1_sgRNA	SETDB1	chr1:150,964,345-150,964,364	GCAGAGGACGTCTTCTTTAG

Table S2.

Oligonucleotides used in this study
